# Herb-sourced emodin inhibits angiogenesis of breast cancer by targeting VEGFA transcription

**DOI:** 10.7150/thno.43622

**Published:** 2020-05-22

**Authors:** Gengyi Zou, Xiaotong Zhang, Lun Wang, Xiyang Li, Tianyu Xie, Jin Zhao, Jie Yan, Longlong Wang, Haoyu Ye, Shunchang Jiao, Rong Xiang, Yi Shi

**Affiliations:** 1 School of Medicine, Nankai University, Tianjin 300071, China.; 2 Department of Oncology, Chinese PLA General Hospital, Beijing 100853, China.; 3 State Key Laboratory of Biotherapy and Cancer Center, West China Hospital, Sichuan University, and Collaborative Innovation Center for Biotherapy, Chengdu 610041, China.; 4 2011 Project Collaborative Innovation Center for Biotherapy of Ministry of Education, Tianjin 300071, China.

**Keywords:** Emodin/NCOR2/SerRS/tumor angiogenesis/VEGFA

## Abstract

Anti-angiogenesis is an important and promising strategy in cancer therapy. However, the current methods using anti-vascular endothelial growth factor A (VEGFA) antibodies or inhibitors targeting VEGFA receptors are not as efficient as expected partly due to their low efficiencies in blocking VEGFA signaling *in vivo*. Until now, there is still no method to effectively block VEGFA production in cancer cells from the very beginning, i.e., from the transcriptional level. Here, we aimed to find bioactive small molecules to block VEGFA transcription.

**Methods:** We screened our natural compound pool containing 330 small molecules derived from Chinese traditional herbs for small molecules activating the expression of seryl-tRNA synthetase (SerRS), which is a newly identified potent transcriptional repressor of VEGFA, by a cell-based screening system in MDA-MB-231 cell line. The activities of the candidate molecules on regulating SerRS and VEGFA expression were first tested in breast cancer cells. We next investigated the antiangiogenic activity *in vivo* by testing the effects of candidate drugs on the vascular development in zebrafish and by matrigel plug angiogenesis assay in mice. We further examined the antitumor activities of candidate drugs in two triple-negative breast cancer (TNBC)-bearing mouse models. Furthermore, streptavidin-biotin affinity pull-down assay, coimmunoprecipitation assays, docking analysis and chromatin immunoprecipitation were performed to identify the direct targets of candidate drugs.

**Results:** We identified emodin that could greatly increase SerRS expression in TNBC cells, consequently reducing VEGFA transcription. Emodin potently inhibited vascular development of zebrafish and blocked tumor angiogenesis in TNBC-bearing mice, greatly improving the survival. We also identified nuclear receptor corepressor 2 (NCOR2) to be the direct target of emodin. Once bound by emodin, NCOR2 got released from SerRS promoter, resulting in the activation of SerRS expression and eventually the suppression of VEGFA transcription.

**Conclusion:** We discovered a herb-sourced small molecule emodin with the potential for the therapy of TNBC by targeting transcriptional regulators NCOR2 and SerRS to suppress VEGFA transcription and tumor angiogenesis.

## Introduction

By transporting nutrients, oxygen and even escaping cancer cells, vascular development plays essential roles in tumor growth and metastasis. Inhibiting tumor angiogenesis keeps an attractive strategy in cancer therapy 1. Vascular endothelial growth factor A (VEGFA), a major stimulator of tumor angiogenesis, as well as vascular endothelial growth factor receptor (VEGFR), are found to be overexpressed in most solid tumors 2,3. Based on these facts, several anti-angiogenic agents have been developed to target either VEGFA or VEGFR 4-6. However, small molecular VEGFR inhibitors cannot block all VEGF receptors which are also able to be upregulated alternatively in solid tumors, dampening the anti-tumor effects of these agents 2,7. Meanwhile, anti-VEGFA antibody, such as bevacizumab, has too high a molecular weight to penetrate into solid tumor tissues 8, which always have high osmotic pressure 9. Theoretically, small molecules with molecular weight less than 1,000 may achieve a better tissue penetration 10. What's more, several serious side effects of these drugs, such as malignant hypertension and cutaneous, renal, hepatic, and hematological toxicities, always mask the optimum and positive effects of anti-angiogenic therapy 11-13.

Despite these facts, anti-angiogenesis therapy keeps applied in clinic for triple negative breast cancer (TNBC), accounting for 15-20% of breast cancer incidence and disproportionally associated with the majority of deaths in breast cancer 14. Lacking ER, PR, and HER2, TNBC has a poor prognosis due to its high aggressiveness, rapid metastases and lack of targeted therapies 15. Bevacizumab, is the only antiangiogenic medicine approved for TNBC by NCCN Clinical Practice Guidelines in Oncology. Randomized clinical trials involved with metastatic breast cancer document that the addition of bevacizumab moderately expands time to progression and response rates, however, has little effect on overall survival due to its limitation of blocking VEGFA expression 16,17. Therefore, development of more efficient antiangiogenic medicines, particularly small compounds with low toxicity, is an urgent priority for TNBC patients. We previously identified a potent transcriptional repressor of VEGFA, i.e., seryl-tRNA synthetase (SerRS) 18, 19. SerRS has been found to compete strongly with c-Myc on the promoter of *VEGFA* in higher vertebrates from fish to human 19. In addition, SerRS can bind directly on telomere to trigger telomere shortening and consequently the senescence of tumor cells 20, manifesting SerRS as a perfect target for cancer therapy.

Traditional Chinese medicine (TCM)-derived small compounds have been demonstrated to have numerous valuable pharmacological activities with low toxicity after their applications in the treatment of many diseases for over a thousand years in Asia 21. Taking these advantages, we have established an in-house library containing 330 herb-sourced small compounds for further screening compounds with antiangiogenic activities by targeting the SerRS-VEGFA pathway. We got a *Rheum palmatum*-derived small molecule, i.e., emodin*. Rheum palmatum* is a widely used Chinese medicinal herb that has been pronounced to have the potential for cancer therapy. Emodin is among the promising active ingredients in *Rheum palmatum* and therefore is involved in our small natural compound library. Emodin is a natural anthraquinone derivative with chemopreventive and chemotherapeutic potential 22. Moreover, previous studies noted the importance of emodin in differentiation-based therapy of cancer cells 23,24. Further investigations are required to gain a better understanding of the possible anticancer properties about emodin. Nonetheless, the direct cellular target of emodin and its biological impacts remain largely unknown. In this study, we revealed a potent anti-angiogenesis activity of emodin in fish model and TNBC mouse models. We also identified nuclear receptor corepressor 2 (NCOR2), which can be recruited by retinoid hormone receptors for transcriptional silencing, as the direct target of emodin, indicating emodin may also be utilized in NCOR2-related pathologies.

## Materials and Methods

### Cell culture

MDA-MB-231 (human breast cancer cells), 4T1 and 4T1-luciferase (murine breast cancer cells) were cultured in Dulbecco's modified Eagle's medium (DMEM; Biological Industries (BI)) supplemented with 10% fetal bovine serum (FBS; BI) and 1% penicillin/streptomycin (P/S; BI). Cells were maintained at 37 °C in a humidified, 5% CO_2_ incubator.

### Chemicals

Emodin and Emodin-biotin (B-Emodin) were synthesized as described in Supplementary Information. Compounds were aliquoted at a concentration of 60 mM in DMSO and stored at -20 °C.

### Animal studies

Female BALB/c NOD-SCID mice (6-8 weeks) and BALB/c mice (6-8 weeks) were purchased from the SPF Biotechnology Co., Ltd (Beijing, China) and allowed to acclimate for one week before use. All mice were maintained in a pathogen-free animal facility with a 12 h light/dark cycle. All murine care and experiments were performed according to the guidelines approved by the Animal Care and Use Committee at Nankai University (Tianjin, China). For the allograft mouse model, 4T1-luciferase cells (1×10^5^) were injected into the #4 mammary fat pad of the mice. When tumors were palpable, mice were randomly divided into three groups (4 mice/group) and received intraperitoneal administration of different doses of emodin or vehicle every other day. Tumor volume (V) was measured by calipers and calculated by the standard formula: V = length × width^2^/2. Besides caliper measurements, tumor volume was also determined by a Caliper Life Science IVIS Lumina II Imager. Bioluminescence was monitored weekly. For the xenograft mouse model, MDA-MB-231 cells (1×10^6^) were injected into the #2 mammary fat pad of the mice. When tumors were palpable, mice were randomly divided into two groups (6 mice/group) and received intraperitoneal administration of emodin or vehicle every other day. Tumor volume was measured by calipers only. For the survival assay, 4T1 cells (1×10^5^) were injected into the #2 mammary fat pad of the mice. When tumors were palpable, mice were randomly divided into two groups (8 mice/group) and received intraperitoneal administration of emodin or vehicle every other day. The mice were monitored regularly for death throughout the whole survival period. The study end point was the time point when all mice in the control group died.

### Dual-Luciferase reporter assay

The promoter region of the SerRS gene was amplified by PCR and cloned into the pGL4.11[luc2P] vector (between Kpn I and Xho I sites) to create the pGL4-pSerRS firefly luciferase reporter plasmid. MDA-MB-231 cells were stably transfected with pGL4-pSerRS firefly luciferase reporter plasmid. Lentivirus carrying renilla luciferase (RenLuc) control reporter plasmid pLV-EF1α-RenLuc (BiOSETTIA, San Diego, CA, USA) was used to stably transfected the MDA-MB-231 cells for normalizing luciferase activity. Luciferase activity was measured by using the Dual-Luciferase Reporter Assay System (Promega, Madison, WI, USA). Primer sequences are in Table S2**.**

### Coimmunoprecipitation assays

MDA-MB-231 cells were treated with emodin for 48 h, then scraped and lysed on ice for 30 min with cell lysis buffer (20 mM Tris-HCl (pH 7.5), 150 mM NaCl, 2 mM EDTA, 1% Triton X100, 1 mM Na_3_VO_4_.12H_2_O) containing 1% protease inhibitor cocktail (Sigma-Aldrich, St. Louis, MO, USA). Cell lysates were incubated with the indicated antibodies (NCOR2, 1:1000, CST 62370; RARα, 1:1000, Abcam ab41934; RXRα, 1:1000, Abcam ab125001) and pre-washed protein-G-agarose beads (CWBIO, Beijing, China) overnight at 4 °C with rotation. Beads were then washed three times with wash buffer (20 mM Tris-HCl (pH 7.5), 150 mM NaCl, 2 mM EDTA, 0.1% Triton X100) and proteins bound on the beads were eluted by boiling in SDS loading buffer (40 mM Tris-HCl pH 6.8, 4% glycerol, 2% SDS, 2 mM Dithiothreiol, 0.01% Bromophenolblue) and separated by SDS-PAGE for western blot to detect NCOR2, RARα and RXRα.

### Western blot

After emodin treatment, MDA-MB-231 and 4T1 cells were lysed with cell lysis buffer (same as previously described) containing 1% protease inhibitor cocktail (Sigma). After blocking with TBST buffer containing 5% milk (BD, San Jose, CA, USA), membranes were incubated with primary antibodies against SerRS (1:1000, made in lab), VEGFA (1:1000, Santa Cruz sc7269), β-actin (1:5000, Santa Cruz sc-47778), NCOR2, ACACA (1:1000, Proteintech 67373-1-Ig), FASN (1:1000, Proteintech 66591-1-Ig), CAD (1:1000, Proteintech 16617-1-AP), α-Tubulin (1:1000, Proteintech 11224-1-AP). All bands were detected using a chemiluminescent substrate ECL kit (Thermo Scientific, Waltham, MA, USA).

### *In vivo* studies in zebrafish

Transgenic Tg (Fli1a: EGFP) fish were raised at 28.5 °C under a 10/14 h dark/light cycle. The night before emodin treatment, male and female zebrafish were kept in the tank containing a fish mating cage (including an inner mesh and divider). Embryos were collected after natural spawning then rinsed with system water. The embryos or larvae were kept at 28.5 °C and incubated in E3 embryo medium (5 mM NaCl, 0.17 mM KCl, 0.33 mM CaCl_2_, and 0.33 mM MgSO_4_, pH 7.2). At 12 h post fertilization, the medium was supplemented with 0.003% 1-phenyl-2-thiourea (PTU) at 28.5 °C to prevent pigment formation. After 72 h post fertilization, larvae were anesthetized with 0.168 mg/mL tricaine (Sigma-Aldrich), and photographed with a DP72 digital camera mounted on an SZX16 dissecting microscope (Olympus Corporation). All zebrafish experiments were approved by the Institutional Animal Care Committee of Nankai University and conformed to the National Institutes of Health Guidelines. The effects of emodin on ISV development were analyzed by χ^2^-test.

### Drug toxicity assay

After treatment with emodin (100 mg/kg body weight) or DMSO for 28 days, BALB/c mice (6-8 weeks) were sacrificed, then blood was drawn from the inner canthus and the major organs were dissected. HE staining and routine blood examinations were performed to analyze the toxicity of emodin. Blood was analyzed by a hematology analyzer (Celltac E, Nihon Kohden).

### Streptavidin-biotin affinity pull-down assay

MDA-MB-231 cells were lysed with cell lysis buffer (same as previously described) containing 1% protease inhibitor cocktail (Sigma). Cell lysates were incubated with free biotin (MedChemExpress, MCE, NJ, USA) or emodin-biotin (10 μM) for 5 h at 4 °C with rotation. Subsequently, the pre-washed streptavidin agarose beads (Yeasen Biotech, Shanghai, China) were added to the system as above and incubated overnight at 4 °C with rotation. The beads were washed three times with elution buffer then denatured protein was separated by SDS-PAGE and visualized by Coomassie blue staining. The indicated bands were excised for mass spectrometry (BGI, Beijing, China).

### Immunofluorescence assay

Paraffin sections of Matrigel plugs were stained with primary antibody against CD31 (1:50, Abcam ab28364) at 4 °C overnight. Subsequently, the slides were incubated with goat-anti-rabbit antibody conjugated Alexa 594 (ZSGB-BIO, Beijing, China) for 1 h at room temperature. Nuclei were detected with DAPI. Slides were photographed by an FV-1000 laser scanning confocal biological microscope (Olympus).

### Hematoxylin and eosin (H&E) staining and immunohistochemical staining

Paraffin sections of tumors and organs were stained with hematoxylin and eosin (H&E) for overall morphological observations. Moreover, tumors were stained with primary antibodies including for SerRS (1:500), VEGFA (1:50), CD31 (1:50), Ki67 (1:200, Abcam ab16667), cleaved caspase-3 (CC3, 1:200, CST 9661), P16 (1:50, Proteintech 10883-1-AP), P21 (1:50, Proteintech 10355-1-AP), β-Gal (1:50, Proteintech 15518-1-AP), iNOS (1:1000, Proteintech 18985-1-AP) and Arg1 (1:1000, Proteintech 160011-1-AP) for immunohistochemical analysis. Sections were observed under a light microscope (Olympus) and the number of positive cells quantified by Image J software v1.50 (100). Sections were scored by counting the number of cancer cells expressing the proteins as determined by CD31 and CC3 staining in tumors. Other markers were measured by H-score. The intensity and proportion scores were then multiplied to give the semiquantitative H-score. 1%~25%, 26%~50%, 51%~75% and 76%~100% were counted as 1 to 4 points respectively according to the proportion of positive cells. None, weak, medium and strong were counted as 1 to 4 points respectively according to the staining intensity.

### Quantitative RT-PCR

Total RNA was extracted from cells using TRIeasy (Yeasen Biotech). Reverse transcription was performed by M-MLV Reverse Transcriptase (Promega). qPCR was performed by a LightCycler 96 (Roche, Basel, Switzerland) with a Hieff qPCR SYBR Green Master Mix (Yeasen Biotech). All primers are listed in Table S2.

### Enzyme-linked immunosorbent assay (ELISA)

After treatment with emodin or DMSO for 48 h, supernatants from cells were collected and 1% protease inhibitor cocktail (Sigma) was added. Supernatants were added to a 96-well microtiter plate pre-coated with an antibody specific to VEGFA (DLDEVELOP, Wuxi, China). Absorbance was measured spectrophotometrically at a wavelength of 450 nm by a microplate reader (Promega). Additionally, total proteins were measured to normalize VEGFA expression levels.

### Matrigel plug angiogenesis assay

1×10^6^ 4T1 cells were mixed with emodin (10 μM) or vehicle and resuspended in 210 μL of a 1:2 mixture of medium:Matrigel (BD Biosciences). BALB/c mice (6-8 weeks) were given subcutaneous injections of this mixture. After seven days, the plug was removed, fixed and stained for CD31 (1:50), VEGFA (1:100) and SerRS (1:500).

### Construction of NCOR2 SANT2 expression vectors

The sequence encoding the SANT2 domain (amino acid residues 427-669) of human NCOR2 proteins was cloned into the BamHI and XhoI sites of pGEX6P-1 (GE Healthcare, MA, USA). Plasmid was transfected into BL21 cells (Cwbio, Beijing, China). Expression of GST-NCOR2 SANT2, recombinant protein was induced by isopropyl 1-thio-β-D-galactopyranoside. The primers used for the plasmid construction are described in Table S2.

### Chromatin immunoprecipitation (ChIP)

ChIP assays were performed using a ChIP-IT Express Enzymatic kit (Active Motif, Carlsbad, CA, USA). Cells were cross-linked with 1% formaldehyde at room temperature for 10 min and the reaction stopped with glycine. After cell fixation and shearing, the supernatant was collected for anti-NCOR2, anti-HDAC3 (1:1000, CST 85057) and anti-RARα immunoprecipitation. Anti-mouse IgG was used as a negative control. ChIPed DNA was analyzed on the QuantStudio 3 (Thermo Fisher Scientific, Waltham, MA, USA) using Hieff qPCR SYBR Green Master Mix (Yeasen Biotech). Two primer sets targeting the RARE on the promoter of SerRS were used. The sequences of the primers are listed in Table S2.

### Statistical analysis

All statistical analyses were performed by GraphPad Prism 5 software (GraphPad Software Inc.) and presented as means ± standard deviation (SD). An unpaired Student's t-test was used for the comparisons between two different groups and ANOVA for larger numbers of groups. A log-rank test was used for the comparisons of survival curves. p values less than 0.05 were considered statistically significant.

## Results

### Emodin inhibits VEGFA expression through promoting SerRS transcription

**Figure 1:**
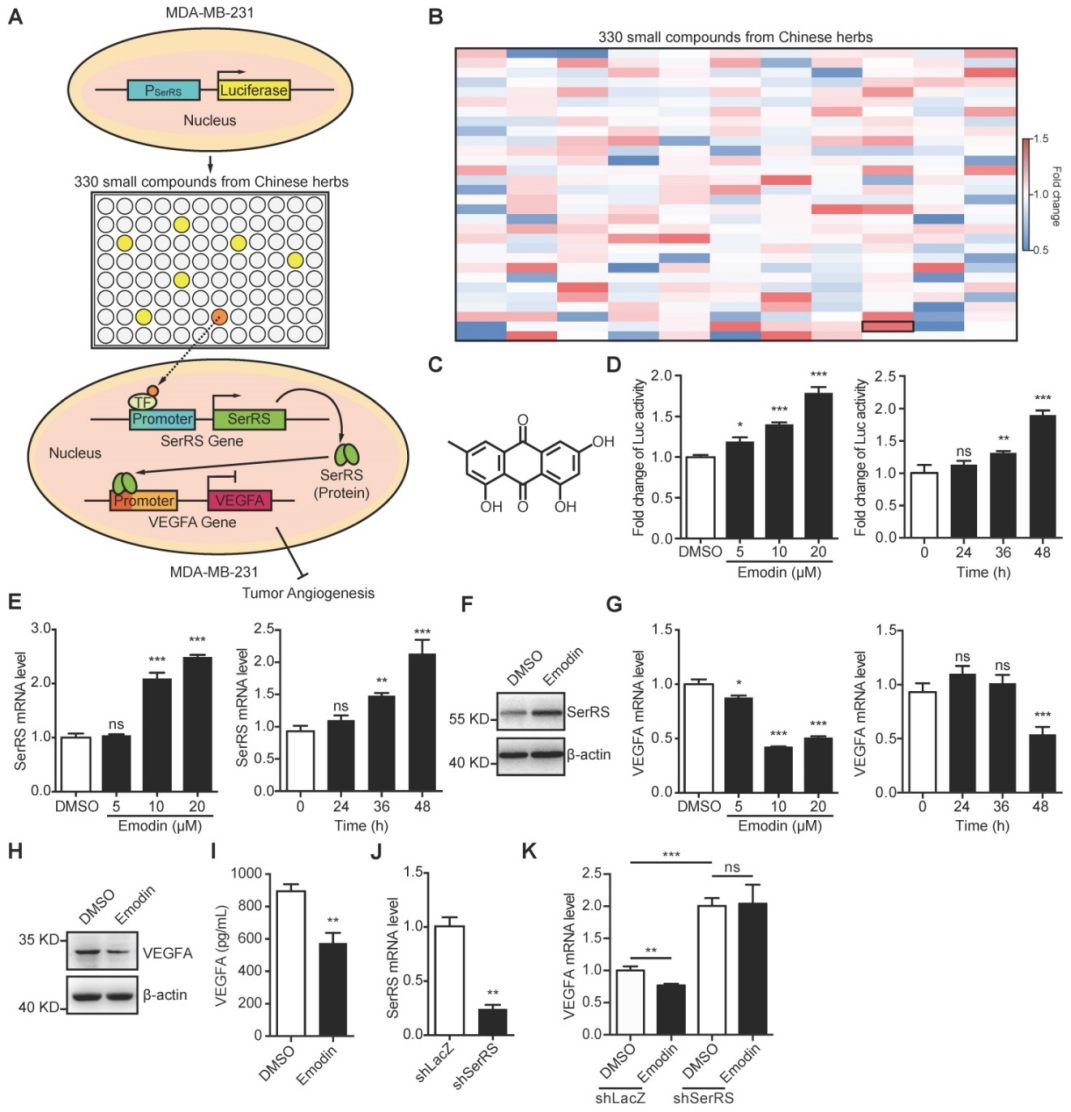
** Emodin increases SerRS expression and suppresses VEGFA expression in MDA-MB-231 cells. (A)** Diagram illustrating system for screening in house library of compounds from Chinese herbs for increased SerRS expression (TF, transcriptional factor). **(B)** Heatmap of 330 compounds that regulate SerRS expression based on firefly/renilla ratio. Compared with DMSO, compounds with >1.0-fold change were regarded as effective compounds. **(C)** Chemical structure of emodin. **(D)** Effect of concentration of emodin and duration of treatment on firefly/renilla ratio. **(E)** mRNA level of SerRS from MDA-MB-231 cells; effect of concentration and treatment time with emodin. **(F)** Protein level of SerRS from MDA-MB-231 cells treated with emodin (10 µM) for 48 h. **(G)** mRNA level of VEGFA from MDA-MB-231 cells; effect of concentration and treatment time with emodin. **(H)** Protein level of VEGFA from MDA-MB-231 cells treated with emodin for 48 h. **(I)** ELISA detection of VEGFA expression in MDA-MB-231 cells treated with emodin for 48 h. **(J)** mRNA level of SerRS in MDA-MB-231 cells following lentiviral infection of shRNA targeting human SerRS. **(K)** mRNA levels of VEGFA from MDA-MB-231-shLacZ and MDA-MB-231-shSerRS cells treated with emodin for 48 h. n=3 for D-K; data are means ± SD., *p<0.05; **p<0.01; ***p<0.001; ns, no significant difference; by one-way ANOVA for D, E, G; by Student's t-test for I-K.

Based on our previous findings that SerRS is a conserved potent transcriptional repressor of VEGFA 19 and increased expression of SerRS could suppress the growth of cervical cancer 20, we hypothesized that increasing SerRS expression could be a valuable strategy to suppress tumor angiogenesis and progression. We established a cell-based screening system using a TNBC cell line MDA-MB-231, which was stably transfected with a firefly luciferase reporter gene driven by SerRS promoter region (-2405 to +25) (**Figure 1A**). Next, we screened 330 small compounds isolated from Chinese traditional herbs using this cell-based system and got 107 compounds that were able to increase the expression of reporter gene over 1.0-fold (**Figure 1B**). Among the high ranked hit compounds (**Figure S1**), emodin showed the highest activity to activate reporter gene in a dose- and time-dependent manner (**Figure 1B-D**). Furthermore, 10 μM of emodin was able to significantly increase both the mRNA and protein levels of endogenous SerRS in MDA-MB-231 cells 48 h post treatment (**Figure 1E-F**). As expected, upon treatment with 10 μM of emodin, the transcription of VEGFA in MDA-MB-231 cells decreased significantly in 48 h (**Figure 1G**), so did the cytoplasmic and secreted VEGFA proteins (**Figure 1H-I**). To further confirm whether emodin-induced decrease of VEGFA expression was caused by activated SerRS expression, we knocked down SerRS expression by shRNA, while a non-specific shRNA against beta-galactosidase lacZ was used as a negative control (**Figure 1J**). The reduction of VEGFA expression by emodin was totally inhibited in SerRS-silenced MDA-MB-231 cells (**Figure 1K**), suggesting that emodin-activated SerRS expression is necessary for blocking VEGFA.

### Emodin inhibits both the vascular development in zebrafish and matrigel plug angiogenesis in mice

**Figure 2:**
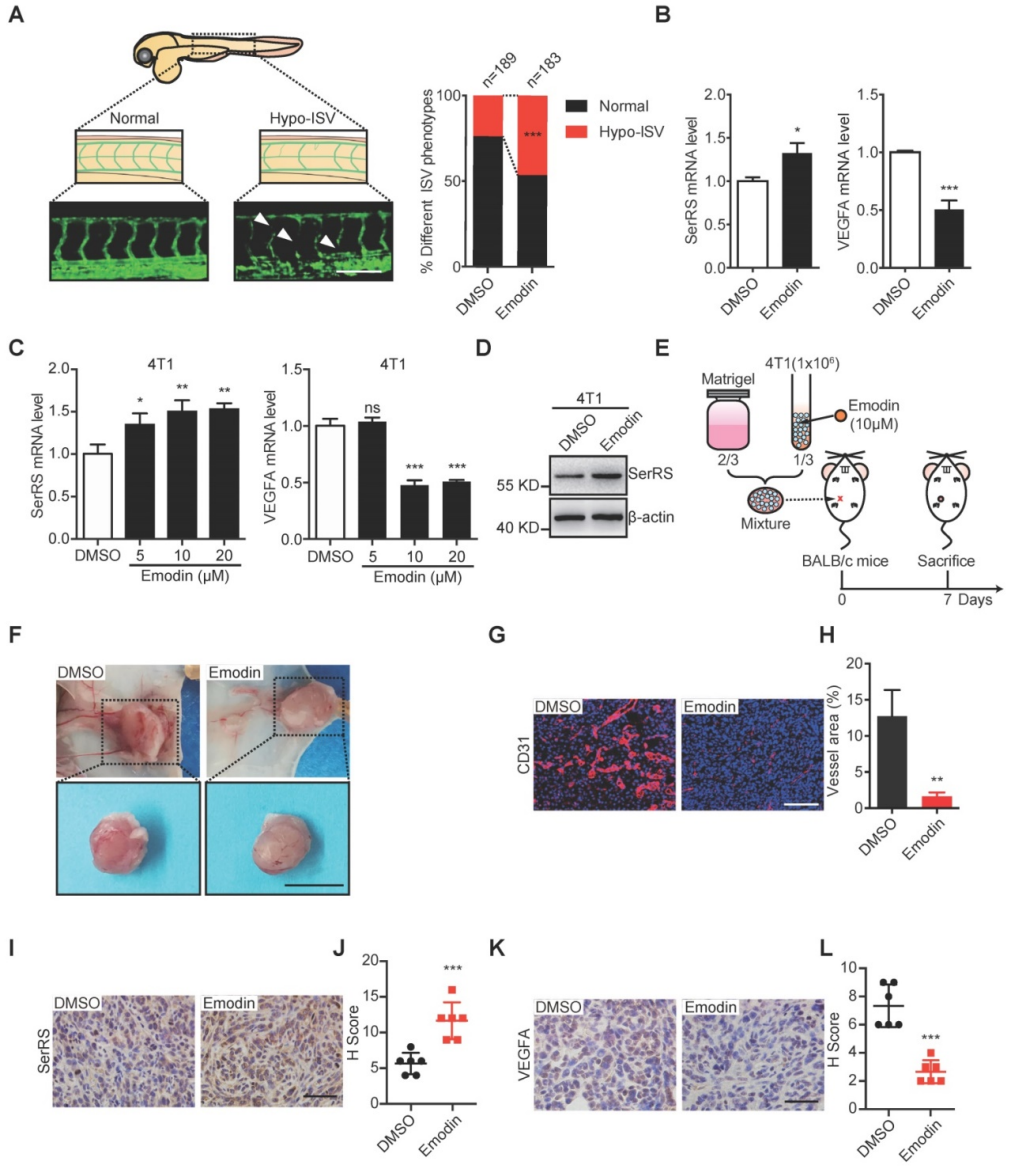
** Emodin inhibits angiogenesis in zebrafish embryos and Matrigel plugs. (A)** Representative Tg (Fli1a: GFP) zebrafish embryos showing normal and hypo (arrowheads) ISV phenotypes at 3 days post fertilization +/- emodin are shown (left); percentage of embryos with each ISV phenotype (right) (Scale bar, 0.2 mm). **(B)** mRNA levels of SerRS and VEGFA from Tg (Fli1a: GFP) zebrafish treated with emodin relative to controls (n=3). **(C)** mRNA levels of SerRS and VEGFA from MDA-MB-231 cells; effect of concentration with emodin. **(D)** Western blot of SerRS expression in 4T1 cells treated with emodin (10 µM) for 48 h (n=3). **(E)** Flowchart of Matrigel plug assay. **(F)** Representative images of Matrigel plugs following vehicle or emodin treatment (n=6). Scale bar, 1 cm. **(G)** CD31 staining (red) in control and emodin-treated Matrigel plugs.** (H)** Percent vessel area in Matrigel plugs. Scale bar, 100 µm. **(I)** SerRS staining of Matrigel plugs. **(J)** Quantification of SerRS-positive cells (n=6). **(K)** VEGFA staining in control and emodin-treated Matrigel plugs. **(L)** Quantification of VEGFA-positive cells (n=6). Scale bar, 100 µm. Scale bar, 100 µm. Data are means ± SD., *p<0.05, ** p<0.01 ***p<0.001; by Chi-square for A; by one-way ANOVA for C; by Student's t-test for B, H, J, L.

To further examine the angiostatic activity of emodin *in vivo*, we utilized transgenic zebrafish (Fli1a:EGFP), which was used previously to study the noncanonical function of SerRS in vascular development 25, to test the effect of emodin on fish angiogenesis at a safe dosage of 10 μM (**Figure S2**). As shown in Figure 2A, emodin significantly inhibited the development of intersegmental vessels (ISV) of zebrafish, with 85 out of 183 (46.4%) emodin-treated zebrafish showing a hypo-ISV phenotype compared with 45 of 189 (23.8%) vehicle-treated fish showing similar phenotype, which totally mimicked the effect of SerRS overexpression in fish embryos 19. Consistently, we observed increased SerRS expression and decreased *Vegfa* expression in emodin-treated fish embryos (**Figure 2B**), suggesting that emodin-caused hypo-ISV phenotype was due to elevated SerRS expression.

To further test whether emodin could inhibit tumor angiogenesis by reducing tumor cell secreted VEGFA, we mixed Matrigel with mouse breast cancer cells 4T1 in the absence or presence of emodin, which could induce SerRS expression and reduce VEGFA expression in 4T1 as well (**Figure 2C-D**), and transplanted the Matrigel plugs subcutaneously into mice (**Figure 2E**). We observed dramatically blocked angiogenesis in emodin-containing plugs (**Figure 2F**) shown by immunohistochemistry (IHC) staining with CD31 antibody (**Figure 2G-H**). IHC staining also showed that emodin significantly increased SerRS expression (**Figure 2I-J**) and reduced VEGFA expression in the matrigel plugs in mice (**Figure 2K-L**). These results confirmed that emodin had robust angiostatic effects *in vivo* by targeting SerRS expression.

### Emodin inhibits the growth of TNBC xenografts in mice

**Figure 3:**
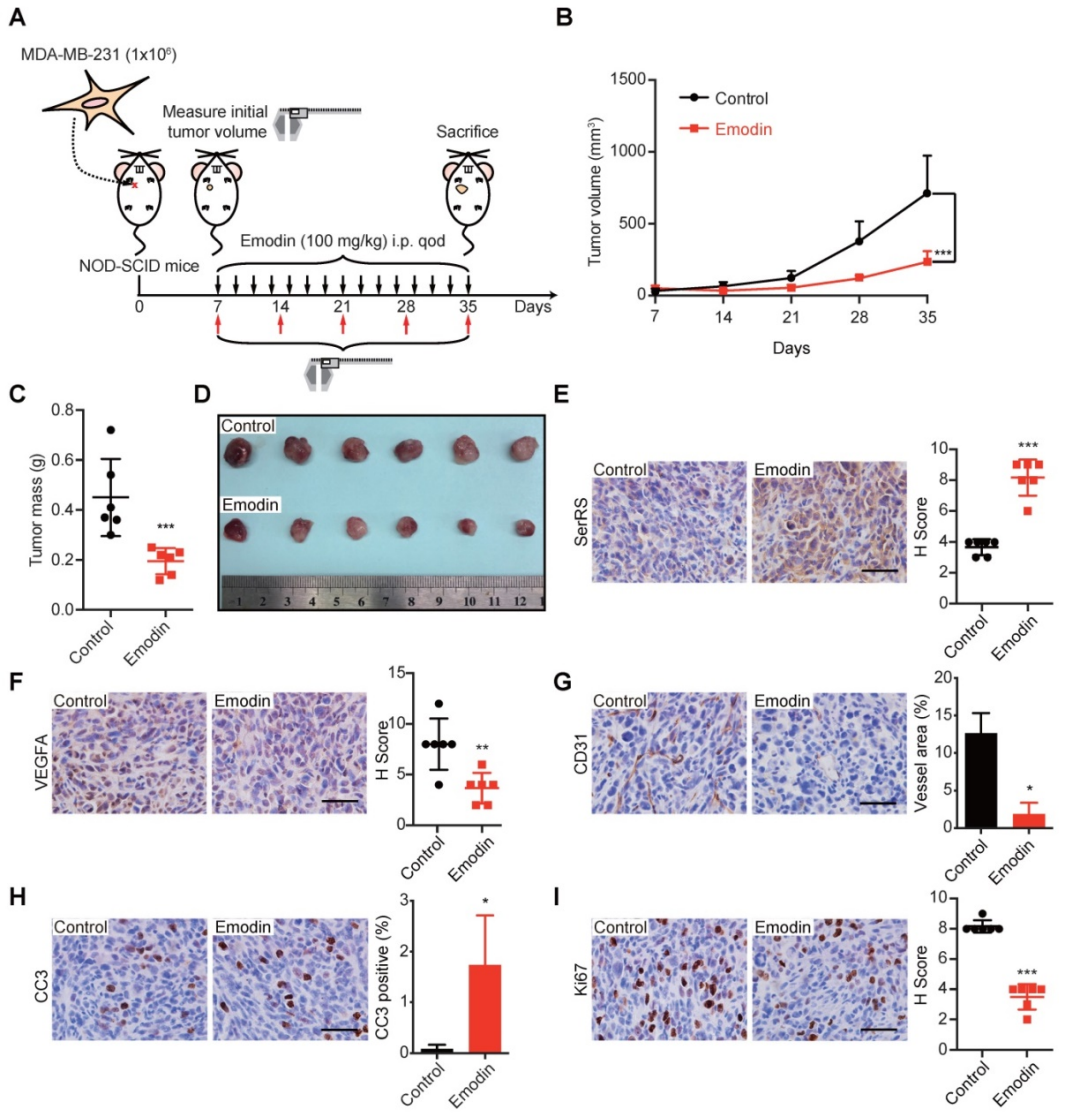
** Emodin exhibits antitumor and anti-angiogenesis activity in a xenograft model. (A)** Flow diagram of MDA-MB-231 cells implanted orthotopically into the 2^nd^ mammary fat pad of female NOD-SCID mice and treated intraperitoneally with DMSO or emodin every other day until sacrifice (n=6). **(B)** Tumor growth curve for control and emodin-treated mice. **(C)** Weight of tumors at sacrifice of the mice. **(D)** Representative images of primary tumors. **(E)** Staining for SerRS-positive cells in control and emodin-treated mouse tumors and quantification (n=6). **(F)** Staining for VEGFA-positive cells in tumors and quantification (n=6). **(G)** CD31 staining of tumors and quantification of vessel area (n=6). **(H)** Staining for Cleaved Caspase 3 (CC3)-positive cells in tumors and quantification (n=6). **(I)** Staining for Ki67-positive cells in tumors and quantification (n=6). Scale bars, 100 µm. Data are means ± SD., *p<0.05, **p < 0.01, ***p < 0.001 by Student's t-test.

Next, we investigated the effect of emodin in the progression of TNBC in MDA-MB-231 xenograft mouse models, which were frequently used to predict the performance of anti-cancer drugs in Phase II clinical trials 26,27. MDA-MB-231 cells were injected subcutaneously into the female immunodeficient NOD-SCID mice 7 days before the administration of 100 mg/kg emodin every other day (**Figure 3A**). We observed a dramatically decreased tumor growth upon emodin treatment (**Figure 3B**). The tumor mass was also measured after the mice were sacrificed 35 days post-inoculation, showing emodin-treated tumors were 2-fold smaller than that in the control group (**Figure 3C-D**). IHC staining showed that emodin was able to promote SerRS expression in tumor tissues (**Figure 3E**) and decrease VEGFA expression (**Figure 3F**), resulting in the greatly blocked tumor angiogenesis shown by CD31 staining (**Figure 3G**). Moreover, emodin-treated tumors showed elevated levels of cleaved caspase-3 (CC3) (**Figure 3H**), indicating that blocked angiogenesis triggered more cell apoptosis. We also tested cell proliferation that could be inhibited by limited nutrient supply due to blocked tumor angiogenesis. As shown in Figure 3I, emodin treatment significantly reduced Ki-67, a well-established cell proliferation marker. Taken together, these results strongly suggested that emodin could efficiently inhibit tumor angiogenesis and hence suppress the growth of TNBC in mice.

To exclude the possibility that the effect of emodin on tumor growth was due to its toxicity on mice, we also measured the weight of the mice upon emodin treatment every week and found no significant weight changes (**Figure S3A**). Also, administration of emodin caused no differences in a variety of blood parameters (**Table 1**) and no toxicity to major organs when evaluated by H&E staining of heart, liver, spleen, lung, kidney and brain (**Figure S3B**).

### Emodin inhibits tumor growth and metastasis in a syngeneic murine TNBC model

**Figure 4:**
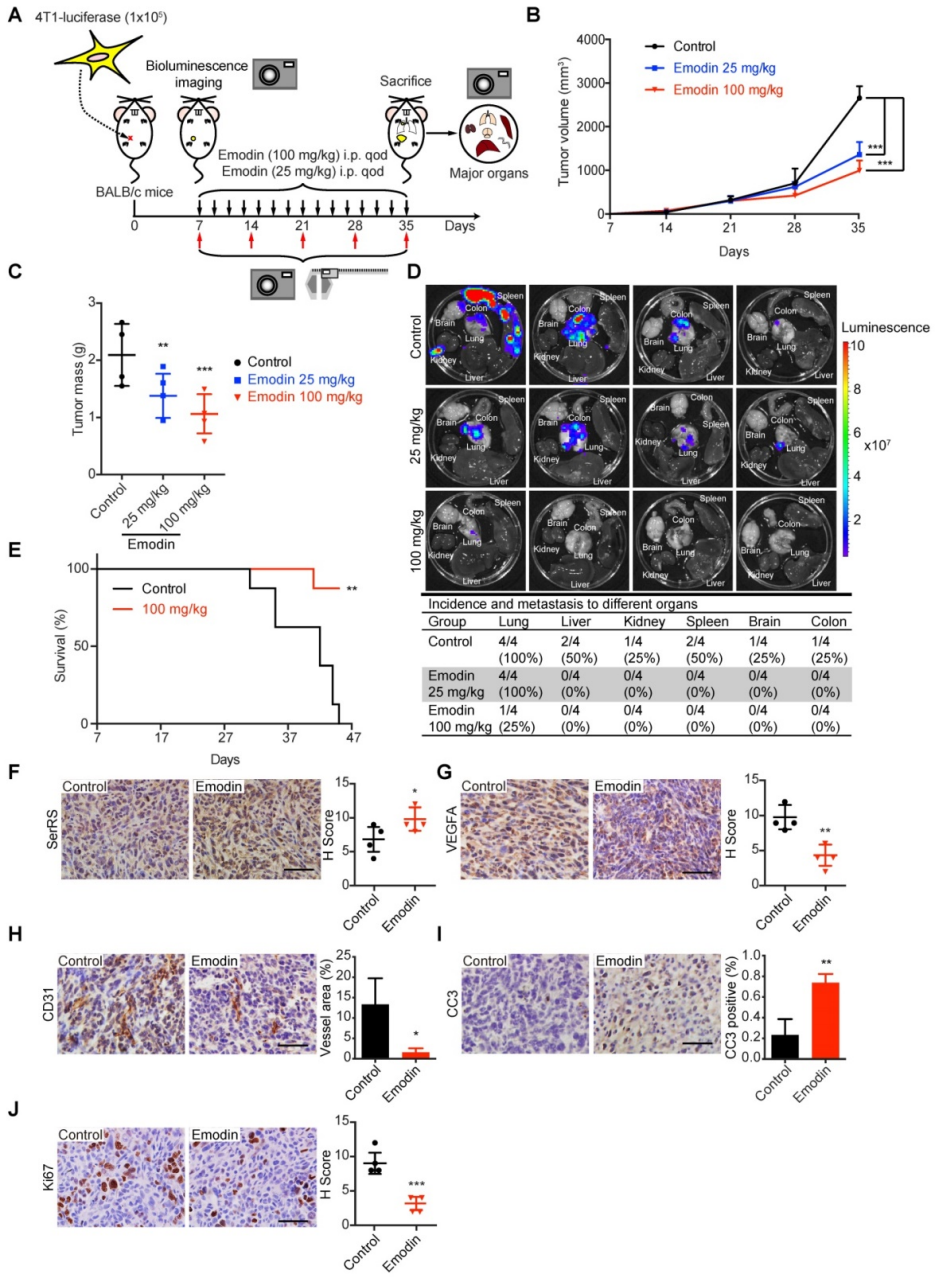
** Emodin inhibits angiogenesis and distant metastasis in an immunocompetent allograft model. (A)** Experimental scheme of 4T1-luciferase cells implanted orthotopically into the 4^th^ mammary fat pad of female BALB/c mice and treated intraperitoneally with DMSO or emodin every other day until sacrifice (n=4). **(B)** Tumor growth curve for control and emodin-treated mice. **(C)** Tumor mass at sacrifice. **(D)** Bioluminescence in major organs indicating the presence of tumors from metastases from the primary tumor and percent incidence in each organ. **(E)** Overall survival rate of mice in different treatment groups (n=8). **(F-J)** Immunostaining of cells and quantification in control and emodin-treated mice for SerRS **(F)**; VEGFA **(G)**; CD31 **(H)**; CC3 **(I)**; and, Ki67 **(J)**. n=4; data are means ± SD., * p<0.05, ** p<0.01, *** p<0.001; by one-way ANOVA for B and C; by Log-rank test for E; by Student's t-test for F-J. Scale bar, 100 µm.

We further investigated the role of emodin against tumor angiogenesis in immunocompetent BALB/c mice since tumor-associated immune microenvironment has been suggested to affect tumor angiogenesis 28. Highly metastatic murine breast cancer cells 4T1, which were stably transfected with luciferase reporter gene, were injected subcutaneously into female BALB/c mice. Seven days after inoculation, different dosages of emodin were administered every other day and the tumor growth and metastasis were monitored by bioluminescent *in vivo* imaging (**Figure 4A**). Consistent to our previous results, emodin greatly inhibited the growth of 4T1 homograft in mice (**Figure 4B-C and Figure S4A-B**). We also examined the metastases of 4T1 cells by measuring the bioluminescence in different organs of the mice. We observed very little 4T1 metastases in the major organs of emodin-treated mice, while in the control group 4T1 metastases were detected in many organs including lung, liver, spleen, brain, kidney and colon (**Figure 4D**). Since tumor metastasis is the main reason of cancer-caused death, we also examined the effect of emodin on the survival of the mice with breast cancer. Consistently, 100 mg/kg of emodin greatly improved the survival of 4T1-bearing mice, with 90% of emodin-treated mice keeping alive 45 days post-inoculation, while all mice in the control group being dead (**Figure 4E**).

To investigate whether the role of emodin in inhibiting the progression of 4T1 homograft was through the SerRS-VEGFA axis, we examined the tumor tissues for related genes by IHC, western blot and quantitative RT-PCR. As shown in Figure 4F, Figure S4C and S4D, emodin significantly increased SerRS expression in tumor cells, leading to the repression of *Vegfa* expression (**Figure 4G and Figure S4D**) and consequently attenuated tumor angiogenesis (**Figure 4H**). As expected, emodin-inhibited angiogenesis induced more apoptosis of tumor cells (**Figure 4I**) and blocked cell proliferation (**Figure 4J**).

### Emodin directly interacts with NCOR2

**Figure 5:**
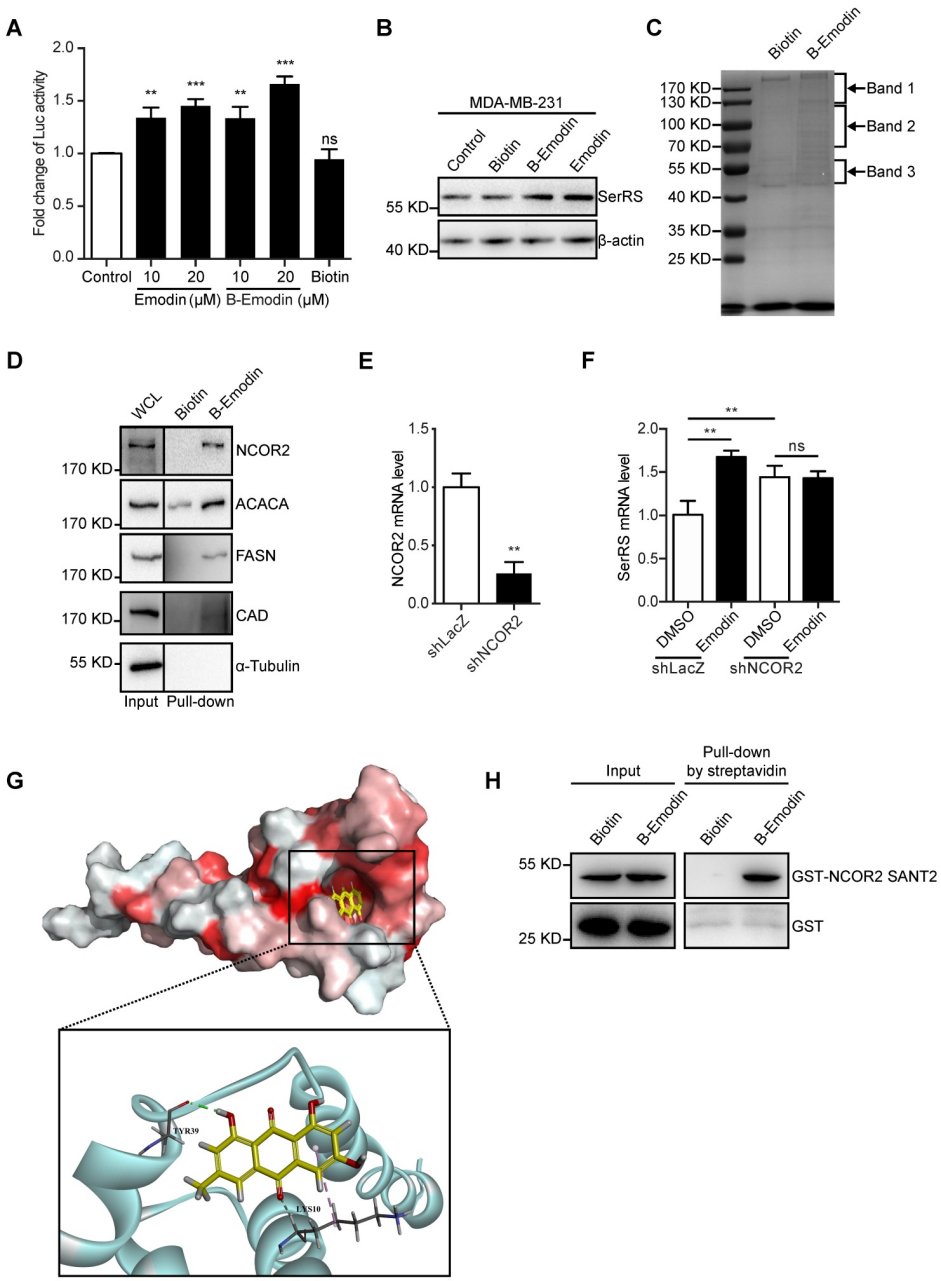
** Emodin can directly bind to NCOR2. (A)** Effect of concentration of emodin and emodin-biotin (B-Emodin) on transcription of SerRS expressed as firefly/renilla ratio (n=3). **(B)** The protein level of SerRS with emodin (10 µM) and B-Emodin (10 µM). **(C)** SDS-PAGE of MDA-MB-231 cell lysates incubated with free biotin or B-Emodin following protein affinity pull-down and coomassie blue staining (n=3). Arrows indicate bands analyzed by mass spectrometry (MS). **(D)** Immunoblot with anti-NCOR2, anti-ACACA, anti-FASN, anti-CAD, and anti-α-Tubulin antibodies of protein precipitated by streptavidin beads from MDA-MB-231 cell lysates in the presence of B-Emodin or biotin (10 µM). **(E)** shRNA target human NCOR2 was transfected into MDA-MB-231 cells, NCOR2 expression in MDA-MB-231 cells was determined by qPCR after shRNA transfection.** (F)** mRNA levels of SerRS from MDA-MB-231-shLacZ and MDA-MB-231-shNCOR2 cells treated with emodin for 48 h (n=3). **(G)** Representations of the predicted binding modes of emodin with NCOR2, SANT2. **(H)** Immunoblot with anti-NCOR2 antibody of purified proteins (GST and GST-NCOR2 SANT2) incubated with B-Emodin (10 µM) or biotin and precipitated by streptavidin beads. Data are means ± SD., ns, no significant difference; **p<0.01; ***p<0.001; by one-way ANOVA for A; by Student's t-test for E, F.

To get insights into how emodin regulates SerRS expression, we next identified the direct target proteins of emodin by purifying emodin-binding proteins from cell lysates with biotin-conjugated emodin (**Figure S5-7**). Biotin-conjugated emodin showed the similar activity as emodin to activate both the expression of luciferase reporter gene driven by SerRS promoter and endogenous SerRS in MDA-MB-231 cells (**Figure 5A-B**). We then incubated lysates of MDA-MB-231 cells with biotin-conjugated emodin followed by affinity purification with streptavidin beads. The specific emodin-associated proteins were resolved by electrophoresis and identified by mass spectrometric (MS) analysis (**Figure 5C and Table S1**). To confirm the MS results, we further examined the interaction between emodin and the cytoplasmic proteins ACACA, FASN, CAD and the nuclear receptor corepressor 2 (NCOR2) which showed relatively high coverage (**Table S1**) by western blot. As shown in Figure 5D, biotin-conjugated emodin could pull down these proteins from the lysates of MDA-MB-231 cells. We were specifically interested in NCOR2, which has been reported to be recruited by RARα/RXRα heterodimer to regulate gene expression 29. Our previous study has shown that retinoic acid receptor RARα/RXRα heterodimer can directly bind on SerRS promoter to enhance its expression once triggered by all-trans retinoic acid 30. In NCOR2-silenced MDA-MB-231 cells (**Figure 5E**), emodin was no longer able to induce SerRS expression (**Figure 5F**), suggesting that NCOR2 was necessary for emodin to regulate SerRS expression.

To further determine whether emodin interacts with NCOR2 directly or indirectly, we firstly performed a docking analysis of emodin into the built-in ligand binding pocket of the SWI3/DAD2N-CoR/TFIII-B2 (SANT2) domain of NCOR2 (PDB ID: 2LTP) by using the Libdock program in the Discovery Studio 3.1 software. The emodin molecule fit in the active pocket of NCOR2 very well. The hydrogen atom on the 3-methylphenol phenolic hydroxyl can form hydrogen bond with tyrosine 39 (Tyr39) to stabilize the binding conformation, and the oxygen atom on the benzoquinone forms a weak hydrogen bond with lysine 10 (Lys10) to enhance the binding of emodin to the NCOR2 (**Figure 5G**). To confirm the direct interaction between emodin and NCOR2, we purified recombinant SANT2 domain of NCOR2 fused with a GST tag and incubated with biotin-conjugated emodin*. In vitro* pull-down assay showed that SANT2 was able to be pulled down with emodin (**Figure 5H**), indicating that emodin directly binds NCOR2. Collectively, our data suggested NCOR2 to be the direct target of emodin.

### Emodin dissociates NCOR2 from the promoter of SerRS

**Figure 6:**
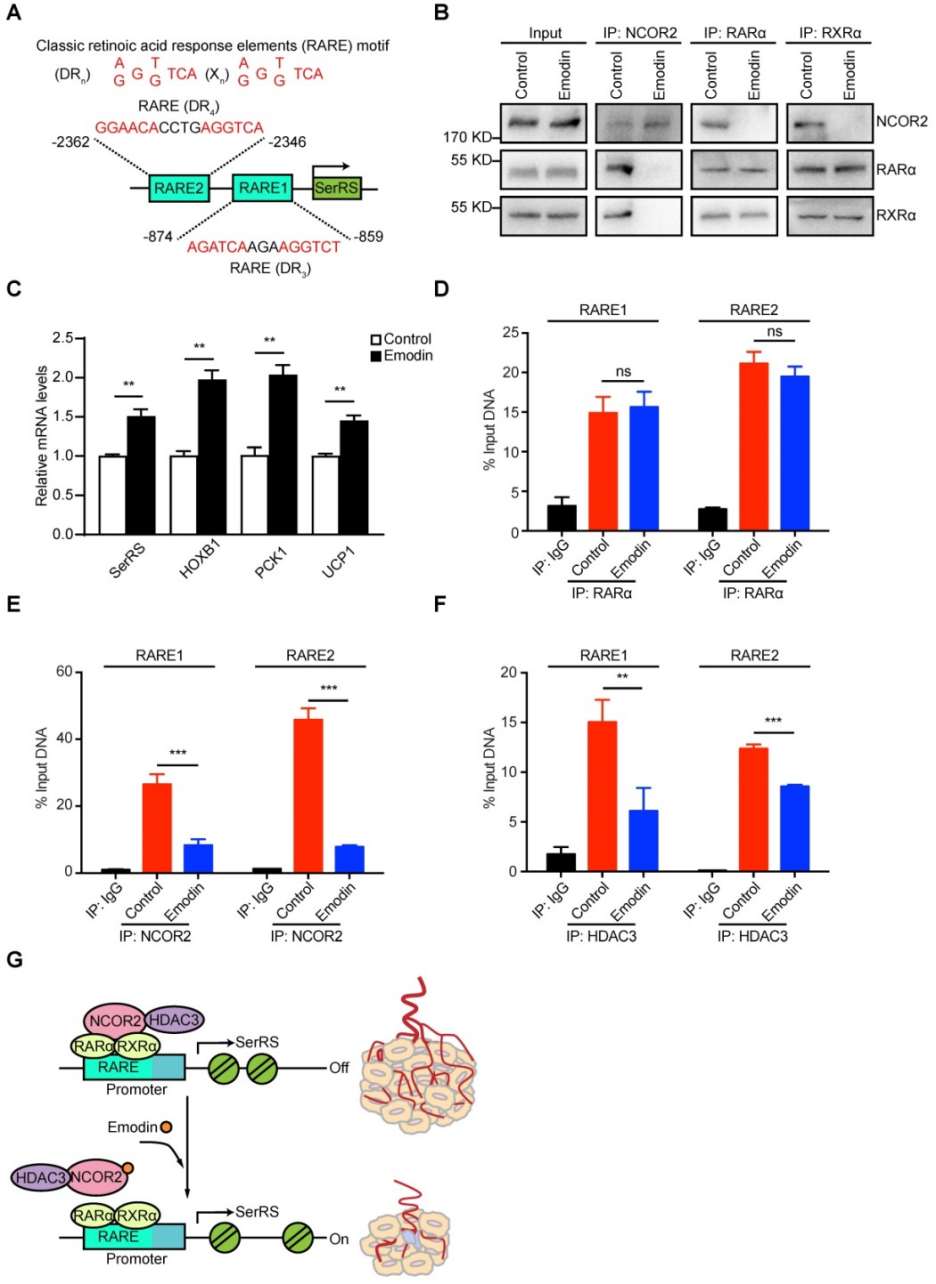
** Emodin can dissociate NCOR2 from the promoter of SerRS. (A)** Two classic retinoic acid response elements (RARE) motifs located on the promoter of SerRS (DR, direct repeats; X, any nucleotide; n, number of interspacing nucleotides). **(B)** Co-immunoprecipitation with anti-NCOR2, anti-RARα and anti-RXRα antibodies of cell lysates after 48 h treatment of MDA-MB-231 cells with DMSO or emodin (10 µM). **(C)** mRNA levels of SerRS, HOXB1, PCK1, and UCP1 from MDA-MB-231 cells treated with emodin (10 µM) relative to controls (n=3).** (D-F)** Chromatin Immunoprecipitation assays of MDA-MB-231 cells with, anti-RARα, anti-NCOR2 and anti-HDAC3 antibodies followed by qPCR with specific primers for the SerRS promoter (n=3; **p<0.01, ***p<0.001, ns, no significant difference; by Student's t-test). **(G)** Model for angiogenesis inhibition by emodin through direct targeting of NCOR2 and subsequent inactivation of the SerRS/VEGFA axis.

NCOR2 acts as a transcriptional corepressor recruited by several nuclear receptors including RARα/RXRα heterodimer 31,32. We previously identified two RARα/RXRα responsive elements (RARE) on SerRS promoter, which can be activated by retinoic acid 30,33 in melanoma cells (**Figure 6A**). We postulated that emodin might interfere with the NCOR2/RARα/RXRα complex to modulate the expression of SerRS. We firstly examined the impact of emodin on the interaction between NCOR2 and RARα/RXRα by co-immunoprecipitation. As shown in Figure 6B and Figure S8, without emodin, both RARα and RXRα were co-immunoprecipitated with NCOR2 antibody as reported 34. In the presence of increased dosages of emodin, NCOR2 was not able to co-immunoprecipitate with either RARα or RXRα any longer, while the interaction between RARα and RXRα was not affected (**Figure 6B and Figure S8**), suggesting that emodin was able to trigger the release of NCOR2 from RARα/RXRα complex in a dose-dependent manner. In addition to SerRS, we also tested the effects of emodin on other well-established downstream targets of RARα/RXRα complex, such as HOXB1, PCK1, and UCP1. As shown in Figure 6C, HOXB1, PCK1, and UCP1 were all upregulated by emodin (**Figure 6C**), confirming that emodin can modulate the transcriptional activity of NCOR2/RARα/RXRα complex to upregulate SerRS expression.

To further study the impact of emodin on the recruitment of NCOR2 on SerRS promoter, we performed chromatin immunoprecipitation (ChIP) followed by quantitative PCR to measure the level of NCOR2 on SerRS promoter in MDA-MB-231 cells. As shown in Figure 6D, we observed the binding of RARα/RXRα on SerRS promoter in MDA-MB-231 cells as well, which was not affected by emodin. We also detected the binding of NCOR2 at the same locations on SerRS promoter (**Figure 6E**), suggesting NCOR2 could be recruited to SerRS promoter by RARα/RXRα. However, in the presence of emodin, the binding of NCOR2 on SerRS promoter was dramatically reduced (**Figure 6E**). NCOR2 functions as a transcriptional corepressor by recruiting histone deacetylase HDAC3 to trigger chromatin condensation and local gene silencing 35. Consistently, we observed a significantly decreased recruitment of HDAC3 on SerRS promoter upon emodin treatment (**Figure 6F**). Taken together, these results suggested that emodin was able to bind NCOR2 directly to dissociate it from the RARα/RXRα complex on the promoter of SerRS, resulting in the reduced recruitment of HDAC3 and opened SerRS promoter conformation to enhance its transcription (**Figure 6G**).

## Discussion

Current anti-angiogenic strategies in cancer therapy, including VEGF neutralization antibodies and small inhibitors of VEGF receptors, are focusing on blocking the VEGF signaling by targeting VEGF protein and specific downstream signaling pathways, respectively. However, due to the limited transportation efficiency of antibodies, in comparison with constant and high production VEGF from tumor cells, and the activation of alternative VEGF downstream signaling, these agents only achieve limited clinical benefits 36,37. Therefore, we hypothesized that blocking the VEGF from the transcriptional level in tumor cells could be a more efficient way. Our previous studies discovered a strong transcriptional repressor of VEGFA, namely SerRS, providing a valuable target for small molecular drugs. We thought targeting SerRS might be a better strategy than targeting VEGFA directly, because SerRS has a broader anti-tumor mechanism besides blocking angiogenesis, for example, SerRS can also promote tumor senescence 20. For targeting method, we chose herb-sourced small molecules due to their better *in vivo* distribution capacity and low toxicity that have been proved in clinical practice, although it remains challenging to get small molecules targeting transcriptional factors. We successfully identified a small natural molecule emodin, which might be the first small molecule that suppresses VEGFA at the transcriptional level. As expected, emodin has robust activity to inhibit angiogenesis in both fish and TNBC in mice, which benefits the survival of tumor-bearing mice.

Emodin is a natural anthraquinone derivative that possesses a wide range of pharmacological properties, particularly the anticancer activity 22. As we mentioned above, triggering telomere shortening and the senescence of tumor cells could be another advantage of SerRS to suppress tumor progression 20. By measuring the cellular senescence markers P16, P21 and β-Gal, we found that emodin can trigger the senescence of breast cancer cells as well (**Figure S9**). Moreover, in accordance with the previous studies, our data demonstrated that in addition to VEGFA targeting emodin had important chemotherapeutic potentials including cytotoxicity and induction of apoptosis (**Figures 3H and 4I**) 38. Another interesting finding is the confirmed association of emodin with macrophages. Besides promoting angiogenesis, it has been reported that tumor associated macrophages (TAMs) could regulate tumor growth and metastasis 39. Both M1 macrophages and M2 macrophages are closely related to inflammatory responses, M1 macrophages are mainly involved in pro-inflammatory responses and M2 macrophages are mainly involved in anti-inflammatory responses 40. As shown in Figure S10, we identified emodin inhibited M2 like TAMs polarization and increased the level of M1-like macrophages. The results indicated that the anti-tumor effect of emodin in 4T1 mouse models was also partly due to its effect on modulating tumor-associated macrophages. Furthermore, macrophage migration inhibitory factor (MIF), which has primarily been known for its proinflammatory effects in autoimmune diseases such as multiple sclerosis and rheumatoid arthritis, has been shown to play important roles in regulating tumor microenvironment 41-44. In several types of cancers, MIF can promote tumor growth by multiple strategies, including promoting angiogenesis via upregulating the secretion of VEGF 45-48. Meanwhile, previous research has indicated the potential of emodin in repressing the expressions of MIF and HIF-1 alpha (HIF-1α) in cervical cancer 49, 50. Since SerRS has been shown to acquire a nuclear function as a transcriptional regulator, it is possible that MIF and HIF-1α are downstream target genes of SerRS in addition to VEGFA. Therefore, further studies are needed to better understand the biological effects and underlying mechanisms of emodin in modulating oncogenic proinflammatory events including the MIF/HIF-1α axis.

Multiple mechanisms for emodin antitumor activity have been explored, but none of them touched its direct target 51-55. In our study, we identified NCOR2 as the direct target of emodin, which bound emodin through its histone-interacting domain SANT2. Thus, we found that emodin performed the anti-angiogenesis function in breast cancer through modulating NCOR2 activity on SerRS promoter. NCOR2 is a transcriptional co-repressor that can assists Nuclear Receptors (NRs) to recruit histone deacetylases, like HDAC3, to DNA promoter regions, downregulating target gene expression 56,57. Recent studies indicated that NCOR2 was involved in multiple disorders ranging from metabolic diseases such as type 2 diabetes to carcinogenesis 58. Elevated levels of NCOR2 have been detected in breast cancer and prostate cancer, promoting cancer progression by suppressing vitamin D3 receptor (VDR) responsiveness 59-61. Multiple tumor-related NRs recruit NCOR2 for their functions, making NCOR2 a valuable target for cancer therapy. Results in the present work reveal emodin as the first NCOR2 inhibitor and provide opportunities for developing NCOR2-targeted agents using emodin as a lead compound. Our study provides the first small molecule against NCOR2, providing a small molecular drug for NCOR2-related pathologies, including cancer.

Two nuclear receptors, RXRs and RARs, has been explored to be drug-targets for tumors and their targeted drugs, like all-trans retinoic acid (ATRA), has been used to treat acute promyelocytic leukemia (APL, APML) for years 62. And our previous study showed that there were RARs and RXRs-responsive elements (RARE) on SerRS promoter, which are responsive to ATRA 30. And now in this research, we revealed that emodin, by targeting NCOR2, could release HDAC3 from RARα/RXRα complex on the promoter of SerRS, finally active SerRS expression 58. Given that NCOR2 are common corepressor for many tumor-related NRs including RARs and RXRs, targeting NCOR2 could have stronger anti-tumor effects than targeting RXR/RAR.

Emodin is a promising antitumor natural product, however; it has a distance for clinical treatment due to its poor pharmacokinetic properties, mainly the low bioavailability, AUC and Cmax 63,64. *In vivo*, emodin rapidly transformed to form its glucuronide via phase II metabolism after intragastric administration 63,64. In further research, we plan to modify the structure of emodin, such as group substitution, to withdraw the problem of phase II metabolism for optimization of the pharmacokinetic properties and even higher antitumor activity.

## Supplementary Material

Supplementary figures and tables.

## Figures and Tables

**Table 1:** Effect of emodin on hemogram results in BALB/c mice

Parameters	Control	Emodin (100 mg/kg)	Reference value
WBC	5.94±1.53	6.48±1.25	4-10
Neutrophil Granulocyte (%)	13.95±1.86	14.74±1.14	45-77
Lymphocyte (%)	95.43±2.35	94.67±1.42	20-40
Monocyte (%)	0.04±0.06	0.06±0.06	0-9
Eosinophilic Granulocyte (%)	0.61±0.51	0.55±0.37	0.5-5
Basophilic Granulocyte (%)	0	0	0-1
RBC	10.6±0.36	11±0.35	3.5-5.5
Hemoglobin	154.33±2.08	153.33±1.53	110-160
Hematocrit	35.27±0.86	35.03±0.46	36-50
MCV	33.4±0.61	31.9±0.53	86-100
MCH	14.57±0.4	13.93±0.32	26-31
MCHC	338±6.08	337.67±4.16	310-370
RDW	13.43±0.25	13.57±0.21	37-50
PLT	280.67±51.6	300±71.57	120-380
PDW	12.3±0.46	12.5±0	9-17
MPV	4.77±0.25	4.67±0.06	9-13
Plateletcrit	0.31±0.04	0.29±0.03	0.12-0.3

All values are expressed as the means ± SD. (n=3 in each group). MCH: mean corpuscular hemoglobin; MCHC: mean corpuscular hemoglobin concentration; MCV, mean corpuscular volume; MPV, mean platelet volume; PDW, platelet distribution width; PLT, blood platelet; RDW, red blood cell distribution width; WBC, white blood cell; RBC, red blood cell.

## Abbreviations

VEGFA: Vascular endothelial growth factor A
VEGFR: vascular endothelial growth factor receptor
TNBC: triple negative breast cancer
SerRS: seryl-tRNA synthetase
TCM: Traditional Chinese medicine
NCOR2: nuclear receptor corepressor 2
TF: transcriptional factor
ISV: intersegmental vessels
IHC: immunohistochemistry
CC3: cleaved caspase-3
MS: mass spectrometric
SANT2: SWI3/DAD2/N-CoR/TFIII-B2
B-Emodin: emodin-biotin
RARE: retinoic acid response elements
ChIP: chromatin immunoprecipitation
NRs: Nuclear Receptors
VDR: vitamin D3 receptor
ATRA: all-trans retinoic acid
