# Supplementary material for: Herb-sourced emodin inhibits angiogenesis of breast cancer by targeting VEGFA transcription
Source: Theranostics. 2020 May 22;10(15):6839–53. doi: 10.7150/thno.43622 (PMC7295066; doi:10.7150/thno.43622)
Supplement: Supplementary file 1 — Supplementary figures and tables. [file thnov10p6839s1.pdf]

Supplementary Figures and legends

Compound (20 μM) #	Structure	Fold change of Luc activity
286	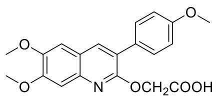	1.48±0.03
376	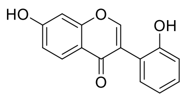	1.46±0.09
82	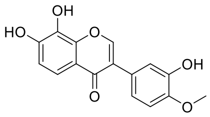	1.43±0.06
352	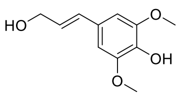	1.43±0.02
16	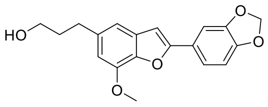	1.43±0.03
223	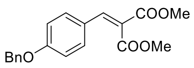	1.40±0.13
164	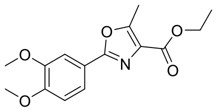	1.40±0.03
382	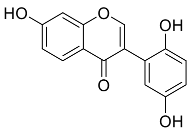	1.40±0.03
117	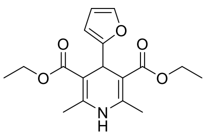	1.40±0.27
188	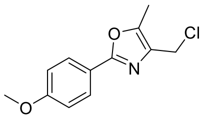	1.40±0.05

Figure S1. High ranked hit chemicals.

Structures of other high ranked hit chemicals from the in-house library and their fold changes of Luc activity on MDA-MB-231 cells (n=3). Data are means ± SD.

	DMSO	Emodin (10 μM)	Emodin (20 μM)
Abnormal morphology	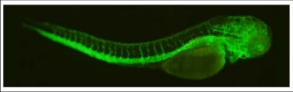	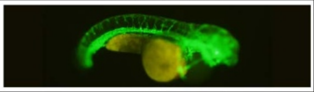	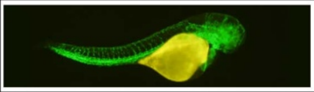
Normal morphology	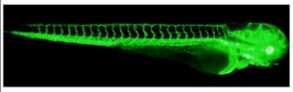	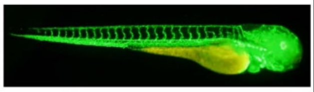	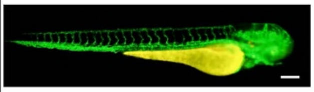
% Abnormal morphology	6% (3/50)	7.7% (4/52)	40.8% (20/49)
P value		0.7354	<0.001

Figure S2. Drug toxicity of emodin in zebrafish model.

Representative normal and abnormal morphologies of Tg (Fli1a: GFP) zebrafish vascular with different concentration of emodin are showed on the 3rd day post fertilization; percentage of abnormal fish vascular morphology in each group was analyzed by $\chi 2$-test (Scale bar, 0.2 mm).

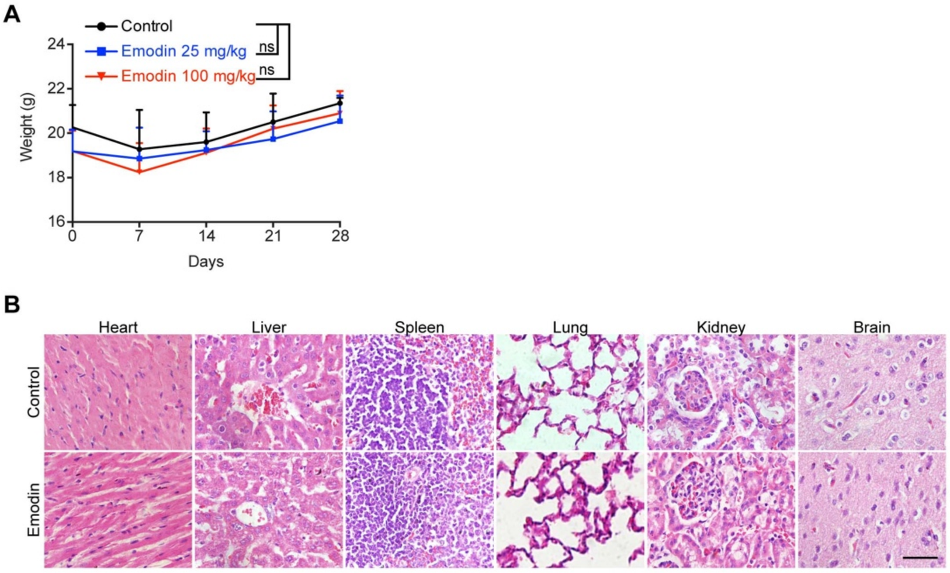

Figure S3. Emodin has low toxicity in a mouse model.

DMSO or emodin was administered intraperitoneally. **(A)** Weight of each mouse monitored weekly (n=4; no significant differences found by one-way ANOVA). **(B)** H&E staining of various organs from control and emodin-treated mice (n=4). Scale bar, 100 μm.

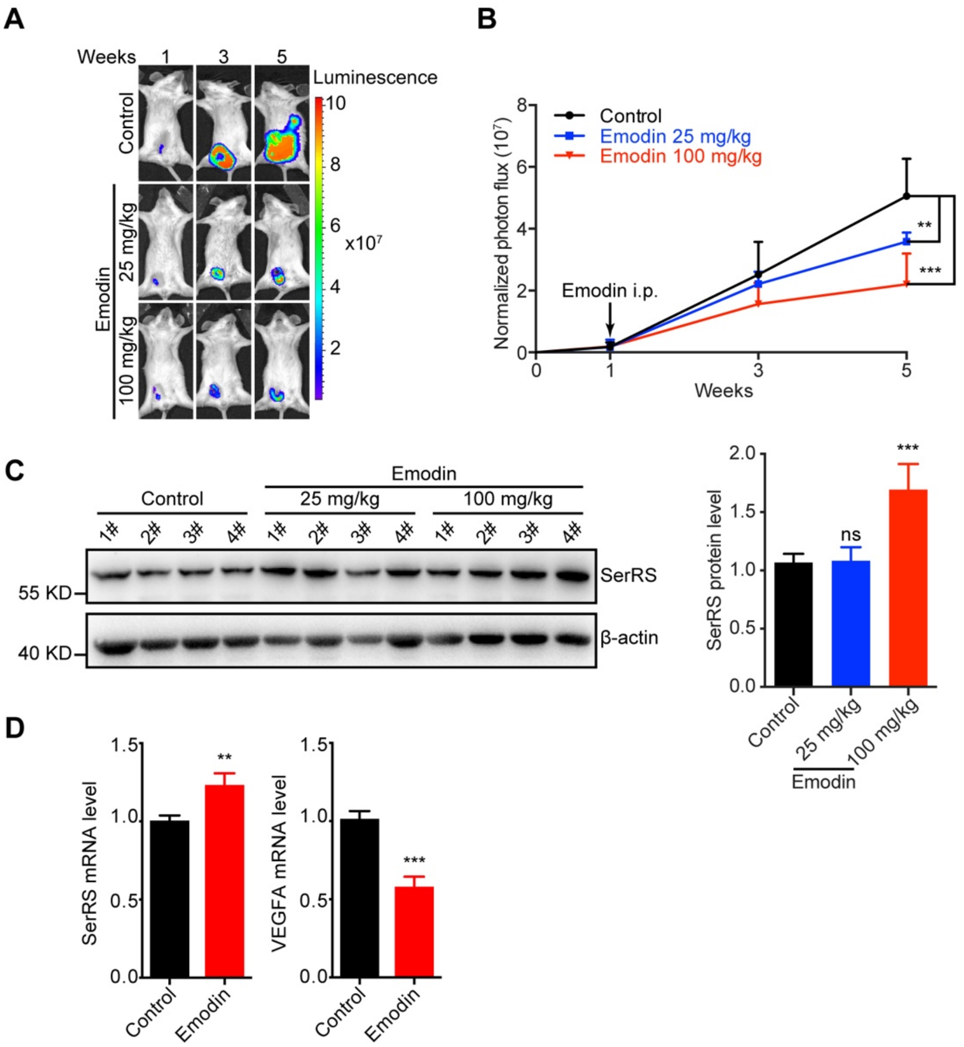

Figure S4. Emodin can inhibit tumor growth in the mouse breast cancer allograft model. (A) Bioluminescence of 4T1-Luciferase cells implanted orthotopically in the 4th mammary fat pad of female BALB/c mice. DMSO or emodin was administered i.p. and growth monitored weekly (n=4). **(B)** Quantification of bioluminescence. **(C)** Western blot of SerRS protein from primary tumors and expressing levels relative to control (n=4). **(D)** Relative expression levels of SerRS and VEGFA mRNA from primary tumors (n=4). Data are means ± SD., **p < 0.01, ***p < 0.001, ns, no significant difference; by one-way ANOVA for B and C; by Student's t-test for D.

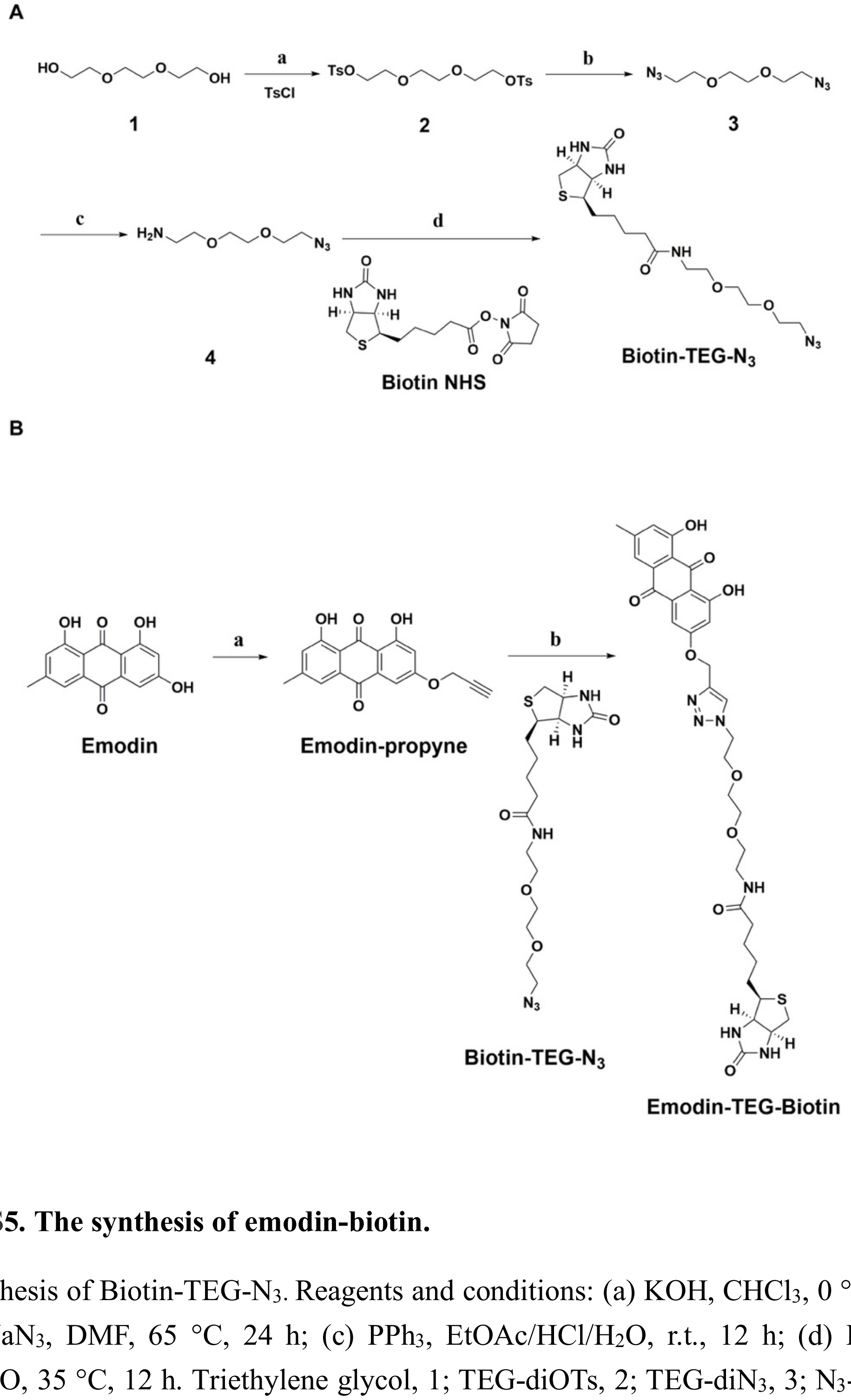

Figure S5. The synthesis of emodin-biotin.

(A) Synthesis of Biotin-TEG-N_3. Reagents and conditions: (a) KOH, $CHCl_3$, 0 °C, 1.5 h; (b) NaN_3, DMF, 65 °C, 24 h; (c) PPh_3, EtOAc/HCl/H_2O, r.t., 12 h; (d) DIEA, DMF/H_2O, 35 °C, 12 h. Triethylene glycol, 1; TEG-diOTs, 2; TEG-diN_3, 3; N_3-TEG-NH_2, 4. **(B)** Scheme of the synthesis of Emodin-TEG-Biotin. Reagents and conditions: (a) K_2CO_3, DMF, 0 °C, 8 h; (b) Et_3N, $CuSO_4$, Click buffer, 35 °C, 5 h.

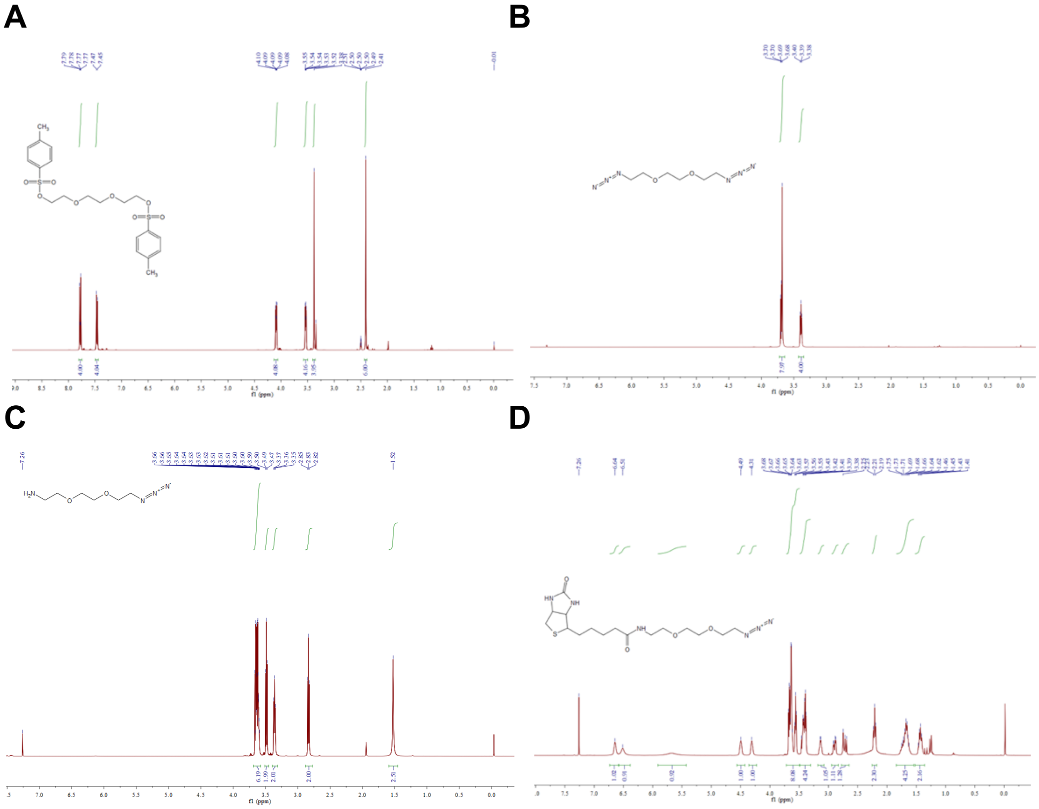

Figure S6. (A) The ^{1}H NMR of TEG-diOTs. ^{1}H NMR (400 MHz, DMSO-d_6) δ 7.81 – 7.75 (m, 4H), 7.46 (d, J = 8.0 Hz, 4H), 4.13 – 4.06 (m, 4H), 3.58 – 3.50 (m, 4H), 3.38 (s, 4H), 2.41 (s, 6H). Exact mass calcd for $C_{20}H_{26}O_8S_2$ $[M+H]^+$: 459.1147; found 459.1150. **(B)** The ^{1}H NMR of TEG-diN_3. ^{1}H NMR (400 MHz, $CDCl_3$) δ 3.72 – 3.64 (m, 8H), 3.42 – 3.35 (m, 4H). Exact mass calcd for $C_6H_{12}N_6O_2$ $[M+H]^+$: 201.1100; found 201.1102. **(C)** The ^{1}H NMR of N_3-TEG-NH_2. ^{1}H NMR (400 MHz, $CDCl_3$) δ 3.69 – 3.57 (m, 6H), 3.49 (t, J = 5.2 Hz, 2H), 3.40 – 3.31 (m, 2H), 2.83 (t, J = 5.2 Hz, 2H), 1.52 (s, 2H). Exact mass calcd for $C_6H_{14}N_4O_2$ $[M+H]^+$: 175.1195; found 175.1193. **(D)** The ^{1}H NMR of Biotin-TEG-N_3. ^{1}H NMR (400 MHz, $CDCl_3$) δ 6.64 (s, 1H), 6.51 (s, 1H), 5.91 – 5.43 (m, 1H), 4.49 (s, 1H), 4.31 (s, 1H), 3.72 – 3.49 (m, 8H), 3.42 (dt, J = 9.9, 7.9 Hz, 4H), 3.13 (d, J = 4.3 Hz, 1H), 2.89 (dd, J = 12.8, 4.5 Hz, 1H), 2.78 – 2.65 (m, 1H), 2.21 (t, J = 7.3 Hz, 2H), 1.69 (ddt, J = 21.0, 13.7, 7.0 Hz, 4H), 1.44 (dd, J = 14.6, 7.3 Hz, 2H). Exact mass calcd for $C_{16}H_{28}N_6O_4S$ $[M+H]^+$: 401.1971; found 401.1970.

A **B**

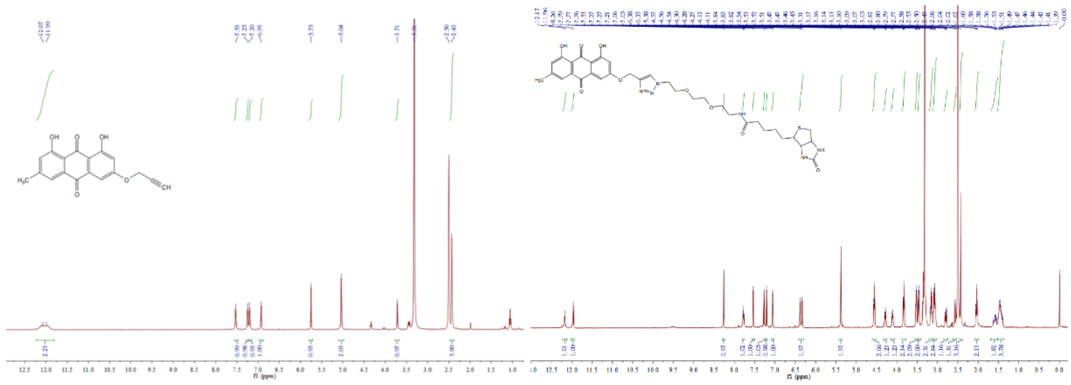

Figure S7. (A) The ^{1}H NMR of Emodin-propyne. ^{1}H NMR (400 MHz, DMSO-d_6) δ 12.03 (d, J = 31.1 Hz, 2H), 7.53 (s, 1H), 7.25 (s, 1H), 7.20 (s, 1H), 6.93 (s, 1H), 5.75 (s, 1H), 5.04 (s, 2H), 3.71 (s, 1H), 2.43 (s, 3H). Exact mass calcd for $C_{18}H_{12}O_5$ $[M+H]^+$: 309.0763; found 309.0765. **(B)** The ^{1}H NMR of Emodin-TEG-Biotin. ^{1}H NMR (400 MHz, DMSO-d_6) δ 12.17 (s, 1H), 11.96 (s, 1H), 8.26 (s, 1H), 7.77 (t, J = 5.5 Hz, 1H), 7.53 (s, 1H), 7.27 (d, J = 2.4 Hz, 1H), 7.21 (s, 1H), 7.05 (d, J = 2.3 Hz, 1H), 6.36 (d, J = 20.1 Hz, 2H), 5.38 (s, 2H), 4.56 (t, J = 5.1 Hz, 2H), 4.34 – 4.24 (m, 1H), 4.11 (d, J = 4.8 Hz, 1H), 3.83 (t, J = 5.1 Hz, 2H), 3.52 (dd, J = 5.7, 3.4 Hz, 2H), 3.46 (dd, J = 5.5, 3.2 Hz, 2H), 3.15 (dd, J = 11.7, 5.8 Hz, 2H), 3.08 (m, 3H), 2.80 (dd, J = 12.5, 5.1 Hz, 1H), 2.56 (d, J = 12.5 Hz, 1H), 2.43 (s, 3H), 2.04 (t, J = 7.3 Hz, 2H), 1.58 (m, 2H), 1.53 – 1.36 (m, 4H). Exact mass calcd for $C_{34}H_{40}N_6O_9S$ $[M+H]^+$: 709.2656; found 709.2656.

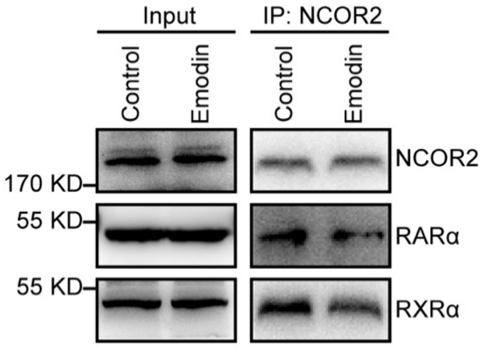

Figure S8. Emodin dissociates NCOR2 from RARα/RXRα heterodimer incompletely.

Co-immunoprecipitation with anti-NCOR2 antibody of cell lysates after 48 h treatment of MDA-MB-231 cells with DMSO or emodin (5 μM).

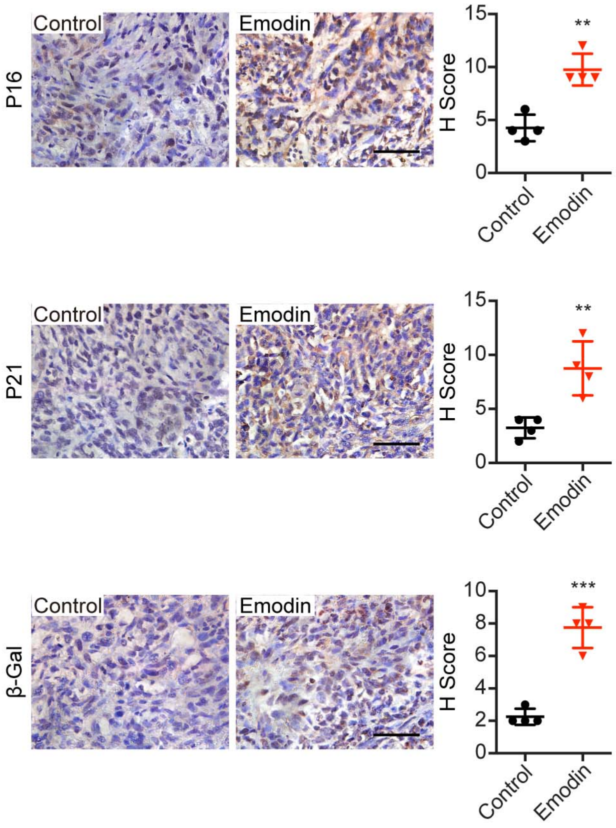

Figure S9. Emodin can promote senescence of tumor cells in the mouse breast cancer allograft model.

Immunostaining of tumor allograft sections from control and emodin-treated mice for senescence markers P16, P21 and β-Gal and the quantification by H-score method (means ± SD, n=4, ** $p<0.01$, *** $p<0.001$ by Student's t-test). Scale bar, 100 μm.

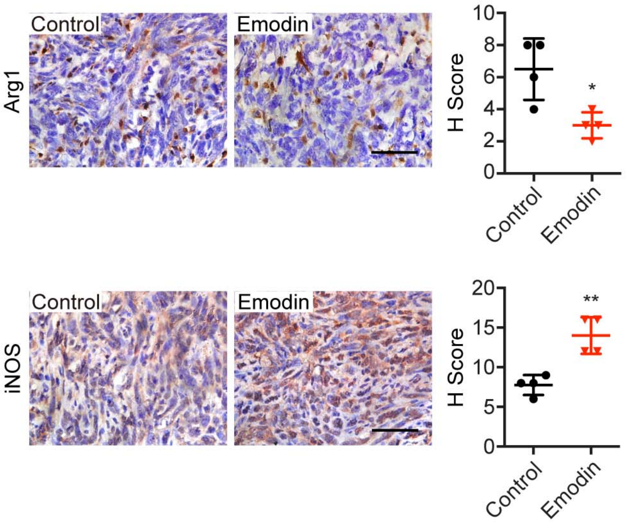

Figure S10. Emodin can regulate the proportion of M1/M2 macrophages. Immunostaining of tumor allograft sections from control and emodin-treated mice for Arg1 and iNOS and the quantification by H-score method (means ± SD, n=4, * $p<0.05$, ** $p<0.01$ by Student's t-test). Scale bar, 100 μm.

Table S1

Band1

Accession	Description	Mass	Score	Matches	Sequences	emPAI	Coverage
Q13085	ACACA	267095	19407	985(674)	129(114)	25.45	60%
O00763	ACACB	278361	3850	204(129)	64(46)	1.37	30%
P35579	MYH9	227646	3722	127(90)	85(67)	3.33	46%
A6NHR9	SMCHD1	227942	2897	133(93)	17(9)	0.49	8%
P04264	KRT1	66170	1871	79(58)	25(25)	4.73	40%
P35527	KRT9	62255	1767	68(45)	26(19)	6.83	46%
Q9NZM1	MYOF	236100	1760	79(56)	65(50)	1.22	40%
P13645	KRT10	59020	1584	51(38)	28(24)	6.85	48%
Q6P2Q9	PRPF8	274738	1368	92(50)	65(44)	0.96	33%
P49327	FASN	275877	1348	61(45)	53(42)	0.8	28%
Q92616	GCN1	294967	1223	66(42)	53(38)	0.64	23%
P27708	CAD	245167	1090	56(35)	48(31)	0.63	29%
P35908	KRT2	65678	849	49(30)	26(21)	2.23	43%
P02538	KRT6A	60293	697	41(27)	24(17)	2.77	39%
P11498	PC	130293	674	33(19)	27(17)	0.77	29%
P48668	KRT6C	60273	666	41(26)	24(16)	2.77	39%
P04259	KRT6B	60315	663	41(27)	22(15)	2.58	36%
O75643	SNRNP200	246006	607	23(18)	22(17)	0.27	12%
P13647	KRT5	62568	575	37(26)	21(16)	2.25	32%
P35658	NUP214	214230	536	31(19)	25(19)	0.37	18%
P08779	KRT16	51578	470	24(16)	18(13)	1.86	41%
P15924	DSP	334021	462	42(15)	38(15)	0.25	13%
P35580	MYH10	229827	446	29(13)	25(11)	0.29	15%
P02533	KRT14	51872	418	22(14)	19(13)	1.52	43%
Q9Y490	TLN1	271766	412	24(13)	22(13)	0.17	11%
Q9P2E9	RRBP1	152780	375	19(11)	17(11)	0.32	17%
P42694	HELZ	220601	357	19(11)	18(11)	0.18	11%
Q04637	EIF4G1	176124	308	32(11)	22(9)	0.27	16%
Q04695	KRT17	48361	239	11(7)	10(7)	0.59	17%
P02768	ALB	71317	191	10(6)	9(5)	0.37	13%
P78527	PRKDC	473749	183	22(6)	18(6)	0.07	5%
Q9Y618	NCOR2	275376	147	12(4)	12(4)	0.07	6%
Q15149	PLEC	533462	145	25(4)	24(4)	0.04	5%
Q14980	NUMA1	239199	125	16(4)	12(4)	0.06	6%
P16402	HIST1H1D	22336	119	27(3)	7(3)	0.75	23%
Q9P2R6	RERE	173457	112	7(4)	6(4)	0.1	5%
P81605	DCD	11391	94	2(2)	2(2)	0.69	25%
Q9UKV3	ACIN1	152170	85	2(2)	2(2)	0.04	1%
Q96N67	DOCK7	244289	81	6(2)	6(2)	0.04	3%

Accession	Description	Mass	Score	Matches	Sequences	emPAI	Coverage
Q14498	RBM39	59628	80	3(2)	3(2)	0.11	7%
P04004	VTN	55069	78	5(2)	4(1)	0.12	6%
Q5D862	FLG2	249296	73	1(1)	1(1)	0.01	0%
P62979	RPS27A	18296	69	3(1)	3(1)	0.4	19%
Q7Z6E9	RBBP6	202354	68	9(2)	8(2)	0.03	4%
P02647	APOA1	30759	64	4(1)	3(1)	0.11	14%
Q13428	TCOF1	152243	57	4(1)	4(1)	0.02	2%
A5YKK6	CNOT1	269106	53	9(2)	9(2)	0.02	3%
P68871	HBB	16102	53	2(2)	2(2)	0.47	14%
P0CL83	STAG3L1	24158	52	12(4)	3(1)	0.3	12%
P69905	HBA1	15305	52	5(2)	4(2)	0.82	23%
O60318	MCM3AP	220662	46	5(1)	5(1)	0.01	3%
Q00839	HNRNPU	91269	44	5(1)	5(1)	0.04	6%
Q13459	MYO9B	244846	43	12(1)	11(1)	0.03	6%
Q16695	HIST3H3	15613	42	3(1)	3(1)	0.22	24%
Q13535	ATR	304764	41	5(2)	4(2)	0.02	1%
O95831	AIFM1	67144	39	2(1)	2(1)	0.05	3%
Q14533	KRT81	56832	39	3(1)	3(1)	0.12	5%
Q14008	CKAP5	227062	38	4(1)	4(1)	0.03	2%
P62805	HIST1H4A	11360	37	3(1)	3(1)	0.3	33%
P21675	TAF1	213969	36	8(1)	8(1)	0.02	4%
Q14697	GANAB	107263	35	5(1)	5(1)	0.03	5%
Q9UPQ9	TNRC6B	194739	34	8(0)	8(0)	0.03	6%
Q96M60	FAM227B	60317	32	1(1)	1(1)	0.05	1%
Q01082	SPTBN1	275237	31	12(1)	10(1)	0.05	4%
P27816	MAP4	121443	26	3(0)	2(0)	0.03	2%
Q9NVE7	PANK4	86621	25	3(0)	3(0)	0.04	4%
Q14669	TRIP12	222234	24	2(0)	2(0)	0.01	1%
P48634	PRRC2A	229180	24	8(0)	7(0)	0.01	3%
Q9Y520	PRRC2C	317346	20	5(0)	5(0)	0.01	1%
P00492	HPRT1	24792	17	1(0)	1(0)	0.14	4%
Q5T5Y3	CAMSAP1	179230	17	4(0)	4(0)	0.02	3%
Q9BXD5	NPL	35254	16	6(0)	1(0)	0.09	3%

Band2

Accession	Description	Mass	Score	Matches	Sequences	emPAI	Coverage
P11498	PC	130293	15776	670(446)	58(46)	14.64	58%
Q13085	ACACA	267095	3096	165(105)	81(62)	1.84	42%
A6NHR9	SMCHD1	227942	2604	118(83)	15(10)	0.43	8%
P13645	KRT10	59020	1492	49(36)	29(24)	6.85	48%
Q00839	HNRNPU	91269	1347	62(37)	28(19)	1.98	37%
P04264	KRT1	66170	1266	51(36)	22(19)	2.05	32%
P35527	KRT9	62255	1060	44(31)	20(19)	3.01	40%

Accession	Description	Mass	Score	Matches	Sequences	emPAI	Coverage
Q15029	EFTUD2	110336	1042	45(33)	33(27)	1.95	48%
P35908	KRT2	65678	868	43(25)	27(19)	2.39	41%
Q7L576	CYFIP1	146742	707	46(28)	35(22)	1.02	29%
P53396	ACLY	121674	623	30(18)	24(18)	0.79	27%
O95373	IPO7	120751	526	20(18)	15(14)	0.62	18%
O75400	PRPF40A	109022	506	26(17)	16(14)	0.75	16%
P49756	RBM25	100467	491	23(17)	15(13)	0.9	24%
Q5VTR2	RNF20	114220	479	20(14)	18(14)	0.57	23%
Q86VP6	CAND1	137999	420	21(13)	16(11)	0.39	15%
Q13751	LAMB3	133366	392	19(10)	17(9)	0.31	19%
P43243	MATR3	95078	384	24(11)	18(9)	0.55	24%
Q92900	UPF1	125578	378	27(12)	24(12)	0.43	27%
P05023	ATP1A1	114135	371	17(13)	16(13)	0.57	20%
Q9H0A0	NAT10	116569	337	15(10)	13(8)	0.39	14%
Q7L2E3	DHX30	134938	314	14(9)	14(9)	0.27	13%
Q9BUJ2	HNRNPUL1	96250	296	21(9)	15(8)	0.4	23%
Q96P70	IPO9	116858	287	8(6)	7(6)	0.21	8%
Q1KMD3	HNRNPUL2	85622	286	21(8)	17(7)	0.46	23%
Q15393	SF3B3	136575	253	11(8)	11(8)	0.24	11%
Q15459	SF3A1	88888	252	13(8)	11(7)	0.34	22%
P13647	KRT5	62568	245	30(11)	19(10)	0.95	31%
Q16531	DDB1	128142	236	15(8)	14(8)	0.25	16%
P19013	KRT4	57649	229	14(8)	5(4)	0.32	8%
P18206	VCL	124292	216	13(6)	13(6)	0.23	14%
P33176	KIF5B	110358	215	13(5)	13(5)	0.23	16%
P08779	KRT16	51578	188	11(7)	9(7)	0.54	17%
P16615	ATP2A2	116336	186	15(8)	13(7)	0.36	16%
Q14694	USP10	87707	169	8(7)	7(6)	0.34	11%
P02533	KRT14	51872	166	10(7)	8(7)	0.54	15%
P29590	PML	99143	149	8(4)	8(4)	0.14	10%
O75150	RNF40	114322	143	6(2)	6(2)	0.06	7%
O75146	HIP1R	119999	131	7(4)	7(4)	0.14	7%
P53992	SEC24C	119789	128	9(3)	9(3)	0.11	10%
P78347	GTF2I	112859	117	12(5)	10(5)	0.29	12%
O00410	IPO5	125032	110	3(2)	3(2)	0.08	4%
Q8WVB6	CHTF18	108001	102	6(3)	6(3)	0.09	8%
Q92974	ARHGEF2	112386	101	5(2)	5(2)	0.09	6%
Q86XI2	NCAPG2	132587	100	4(3)	4(3)	0.08	4%
Q9Y5B6	PAXBP1	105195	91	8(3)	6(3)	0.1	8%
Q9BXP5	SRRT	101060	90	6(2)	5(2)	0.07	7%
Q9NZB2	FAM120A	123008	89	5(2)	5(2)	0.08	4%
Q15149	PLEC	533462	88	14(3)	14(3)	0.03	3%

Accession	Description	Mass	Score	Matches	Sequences	emPAI	Coverage
Q14444	CAPRIN1	78489	86	3(2)	3(2)	0.09	5%
P02768	ALB	71317	86	7(4)	6(3)	0.25	9%
Q9Y2L1	DIS3	110017	80	3(1)	3(1)	0.03	3%
P04004	VTN	55069	74	3(2)	2(1)	0.06	4%
Q9HAV4	XPO5	138332	73	12(3)	12(3)	0.07	12%
Q14498	RBM39	59628	63	3(2)	3(2)	0.11	7%
P42704	LRPPRC	159003	63	9(2)	9(2)	0.06	6%
Q8IWX8	CHERP	104150	62	3(2)	3(2)	0.06	3%
P61626	LYZ	16982	61	1(1)	1(1)	0.2	8%
Q7L014	DDX46	117803	60	6(1)	6(1)	0.03	6%
P26006	ITGA3	117735	59	4(2)	4(2)	0.06	3%
P16402	HIST1H1D	22336	58	22(2)	3(2)	0.32	9%
Q9GZR7	DDX24	96899	55	4(1)	4(1)	0.03	6%
Q8IW35	CEP97	97776	54	2(1)	2(1)	0.03	2%
Q08211	DHX9	142181	53	3(1)	3(1)	0.02	3%
P05556	ITGB1	91664	53	3(1)	3(1)	0.04	4%
Q9BSJ8	ESYT1	123293	52	2(1)	2(1)	0.05	2%
P68871	HBB	16102	51	1(1)	1(1)	0.21	8%
Q8TEX9	IPO4	120179	51	5(2)	4(1)	0.08	5%
Q9BQE3	TUBA1C	50548	51	1(1)	1(1)	0.07	3%
P49736	MCM2	102516	50	3(1)	3(1)	0.03	2%
Q9P2J5	LARS	135577	47	4(1)	4(1)	0.02	3%
P69905	HBA1	15305	45	4(1)	3(1)	0.49	16%
P02647	APOA1	30759	45	3(1)	2(1)	0.11	10%
Q5D862	FLG2	249296	45	3(1)	3(1)	0.01	1%
Q5VTE0	EEF1A1P5	50495	44	1(1)	1(1)	0.07	2%
Q13427	PPIG	89077	43	5(1)	5(1)	0.04	7%
Q96RQ3	MCCC1	80935	43	2(2)	2(2)	0.08	4%
P31151	S100A7	11578	42	1(1)	1(1)	0.3	10%
Q9Y6Y8	SEC23IP	111691	42	3(1)	3(1)	0.06	3%
P42285	MTREX	118756	41	2(1)	2(1)	0.03	2%
P05109	S100A8	10885	40	1(1)	1(1)	0.32	11%
Q8TB72	PUM2	114715	40	9(1)	4(1)	0.03	3%
A0AVT1	UBA6	119207	40	3(1)	3(1)	0.03	3%
O95486	SEC24A	120472	38	1(1)	1(1)	0.03	1%
Q6DN03	HIST2H2BC	21971	38	1(1)	1(1)	0.15	4%
P60709	ACTB	42052	38	2(2)	2(2)	0.16	8%
Q05519	SRSF11	53624	33	1(1)	1(1)	0.06	3%
Q8WWM7	ATXN2L	113589	32	6(1)	3(1)	0.03	1%
P07355	ANXA2	38808	31	2(0)	2(0)	0.09	9%
Q86XP3	DDX42	103197	31	6(1)	4(1)	0.03	6%
Q8N5G2	MACO1	76700	30	11(0)	2(0)	0.04	5%

Accession	Description	Mass	Score	Matches	Sequences	emPAI	Coverage
Q14764	MVP	99551	29	5(0)	5(0)	0.07	5%
Q6ZMQ8	AATK	146247	29	1(0)	1(0)	0.02	1%
Q9BRR8	GPATCH1	103567	28	4(0)	4(0)	0.06	3%
Q9HA77	CARS2	62698	28	6(0)	2(0)	0.05	3%
P62979	RPS27A	18296	26	1(0)	1(0)	0.18	5%
O15226	NKRF	78308	24	1(0)	1(0)	0.04	1%
P29317	EPHA2	109679	24	2(0)	2(0)	0.03	1%
Q99569	PKP4	132585	24	9(0)	4(0)	0.02	3%
P35579	MYH9	227646	24	4(0)	4(0)	0.01	3%
Q96KR1	ZFR	118079	23	4(0)	4(0)	0.03	6%
Q99943	AGPAT1	32038	23	1(0)	1(0)	0.1	3%
P55060	CSE1L	111145	22	1(0)	1(0)	0.03	0%
Q92828	CORO2A	60239	19	1(0)	1(0)	0.05	2%
Q6P1X5	TAF2	138366	17	2(0)	2(0)	0.02	1%
Q92576	PHF3	231563	15	5(0)	3(0)	0.01	1%

Band3

Accession	Description	Mass	Score	Matches	Sequences	emPAI	Coverage
P11498	PC	130293	3273	106(82)	37(28)	1.9	42%
A6NHR9	SMCHD1	227942	2297	107(76)	15(8)	0.45	8%
P04264	KRT1	66170	1380	59(42)	24(20)	2.36	36%
P13645	KRT10	59020	1323	53(39)	28(24)	6.85	49%
P35527	KRT9	62255	885	40(27)	24(20)	2.81	39%
P04406	GAPDH	36201	832	34(23)	12(11)	3.84	39%
P63244	RACK1	35511	783	38(28)	17(16)	6.12	61%
P15880	RPS2	31590	662	31(21)	12(10)	3.94	41%
P04259	KRT6B	60315	652	38(26)	22(18)	2.39	37%
P35908	KRT2	65678	649	35(24)	25(18)	1.93	42%
P02538	KRT6A	60293	574	35(23)	22(18)	2.39	37%
P00338	LDHA	36950	572	28(18)	16(11)	5.06	42%
P61247	RPS3A	30154	557	28(18)	15(11)	7.09	51%
P09651	HNRNPA1	38837	555	20(15)	13(10)	3.01	32%
P62424	RPL7A	30148	479	22(13)	13(9)	4.32	37%
P45880	VDAC2	32060	478	20(13)	12(8)	2.26	46%
P08779	KRT16	51578	452	21(14)	17(13)	1.38	41%
P13647	KRT5	62568	446	28(19)	16(14)	1.39	26%
Q53GQ0	HSD17B12	34416	433	16(11)	10(8)	2.63	33%
P62701	RPS4X	29807	414	22(12)	13(8)	2.55	49%
P06493	CDK1	34131	375	23(11)	16(9)	3.85	60%
P62753	RPS6	28834	368	14(11)	8(6)	1.67	25%
P36542	ATP5C1	33032	363	23(13)	11(8)	2.47	36%
Q86V81	ALYREF	26872	359	11(8)	8(5)	1.55	46%
Q13085	ACACA	267095	345	37(12)	19(10)	0.17	11%

Accession	Description	Mass	Score	Matches	Sequences	emPAI	Coverage
Q99623	PHB2	33276	341	19(10)	13(8)	2.78	51%
P63104	YWHAZ	27899	331	10(7)	7(6)	1.76	35%
Q9H9B4	SFXN1	35881	315	10(8)	9(7)	1.03	31%
P62258	YWHAE	29326	309	13(10)	8(8)	2.27	38%
P27348	YWHAQ	28032	306	12(9)	9(8)	2.07	39%
Q00325	SLC25A3	40525	295	29(11)	9(4)	1.19	23%
P67775	PPP2CA	36142	290	13(9)	10(8)	1.62	40%
P13646	KRT13	49900	290	17(12)	13(11)	1.15	25%
P19013	KRT4	57649	288	17(11)	6(3)	0.32	11%
Q00839	HNRNPU	91269	262	11(5)	8(4)	0.24	12%
P02533	KRT14	51872	242	20(11)	17(11)	1.09	43%
P16152	CBR1	30641	220	7(6)	6(5)	0.86	35%
Q15717	ELAVL1	36240	218	9(6)	8(6)	1.02	26%
P04083	ANXA1	38918	218	6(4)	6(4)	0.39	27%
O00299	CLIC1	27248	218	8(6)	7(5)	1.52	44%
P07355	ANXA2	38808	215	10(7)	9(7)	0.92	32%
P12035	KRT3	64549	199	15(8)	13(7)	0.64	16%
Q13011	ECH1	36136	185	7(6)	7(6)	0.69	22%
P60709	ACTB	42052	185	12(7)	7(6)	1.13	29%
Q9UL46	PSME2	27555	175	4(3)	4(3)	0.41	22%
P09661	SNRPA1	28512	167	7(4)	7(4)	0.56	31%
Q96HS1	PGAM5	32213	162	12(7)	10(6)	1.19	36%
P08758	ANXA5	35971	161	8(5)	7(5)	0.7	25%
Q02978	SLC25A11	34211	158	6(4)	5(4)	0.59	18%
P11142	HSPA8	71082	156	6(4)	5(4)	0.2	8%
Q8NBQ5	HSD17B11	33257	151	6(5)	6(5)	0.61	22%
Q9NUQ9	FAM49B	37010	150	4(3)	4(3)	0.41	18%
P62917	RPL8	28235	149	14(4)	8(3)	0.95	36%
Q5VTE0	EEF1A1P5	50495	145	5(5)	4(4)	0.37	9%
Q9HA64	FN3KRP	34618	140	9(5)	7(4)	0.58	24%
P61289	PSME3	29602	133	4(3)	4(3)	0.53	19%
P25786	PSMA1	29822	133	6(4)	6(4)	0.53	29%
P11177	PDHB	39550	132	5(3)	5(3)	0.27	18%
Q14165	MLEC	32385	129	3(3)	3(3)	0.34	13%
Q92688	ANP32B	28941	128	3(3)	3(3)	0.39	15%
Q9HAN9	NMNAT1	32141	128	5(4)	5(4)	0.48	21%
P10412	HIST1H1E	21852	123	9(4)	8(4)	1.35	29%
O14579	COPE	34688	122	5(4)	5(4)	0.58	20%
Q9UNQ2	DIMT1	35499	117	5(4)	5(4)	0.43	17%
P81605	DCD	11391	112	4(4)	3(3)	1.87	25%
Q9BU76	MMTAG2	29679	111	6(4)	5(4)	0.53	22%
P02768	ALB	71317	111	9(3)	8(3)	0.25	11%

Accession	Description	Mass	Score	Matches	Sequences	emPAI	Coverage
Q16762	TST	33636	110	6(4)	6(4)	0.46	28%
Q8NE86	MCU	40127	110	4(1)	4(1)	0.08	13%
Q13825	AUH	35871	108	9(4)	8(4)	0.7	28%
P24534	EEF1B2	24919	107	3(2)	3(2)	0.29	16%
Q9Y6C9	MTCH2	33936	107	7(5)	6(5)	0.59	25%
O15144	ARPC2	34426	105	8(2)	8(2)	0.74	28%
Q9H936	SLC25A22	34904	100	9(3)	8(3)	0.58	22%
Q3SY84	KRT71	57769	100	5(4)	4(3)	0.25	4%
Q8TDX7	NEK7	34985	98	6(4)	4(3)	0.31	14%
P06753	TPM3	32987	95	8(4)	6(4)	0.47	19%
P21796	VDAC1	30868	93	5(2)	4(2)	0.23	14%
Q15785	TOMM34	34937	92	3(2)	3(2)	0.31	13%
Q16629	SRSF7	27578	92	9(3)	8(3)	0.58	31%
P40926	MDH2	35937	89	3(2)	3(2)	0.19	12%
P23396	RPS3	26842	89	4(2)	4(2)	0.26	18%
P69905	HBA1	15305	88	5(3)	4(2)	1.72	23%
Q16795	NDUFA9	42654	87	6(3)	5(3)	0.45	15%
P49756	RBM25	100467	83	3(2)	2(1)	0.07	3%
O14618	CCS	29536	82	2(1)	2(1)	0.11	7%
Q9BVA1	TUBB2B	50377	78	2(2)	2(2)	0.14	6%
P36873	PPP1CC	37701	77	1(1)	1(1)	0.09	5%
Q9H0U3	MAGT1	38411	77	2(1)	2(1)	0.09	7%
P15927	RPA2	29342	77	4(2)	3(1)	0.38	21%
Q8WXX5	DNAJC9	30062	75	3(2)	3(2)	0.23	12%
Q5RKV6	EXOSC6	28503	75	2(2)	2(2)	0.25	11%
P50914	RPL14	23531	74	2(1)	2(1)	0.14	10%
Q66PJ3	ARL6IP4	45287	73	2(2)	2(2)	0.15	7%
Q9BQE3	TUBA1C	50548	73	4(2)	4(2)	0.13	12%
O14828	SCAMP3	38661	68	2(1)	2(1)	0.09	6%
Q13868	EXOSC2	32996	66	3(1)	3(1)	0.21	13%
Q9UBV8	PEF1	30646	66	4(2)	4(2)	0.23	13%
P67936	TPM4	28619	60	6(3)	4(3)	0.39	16%
Q15459	SF3A1	88888	59	1(1)	1(1)	0.04	2%
Q9BWM7	SFXN3	35823	59	3(1)	3(1)	0.19	10%
Q5D862	FLG2	249296	58	1(1)	1(1)	0.01	0%
P61619	SEC61A1	52687	56	3(2)	3(2)	0.2	6%
P68871	HBB	16102	56	3(2)	3(2)	0.77	23%
O75569	PRKRA	34839	55	1(1)	1(1)	0.1	3%
O00487	PSMD14	34726	54	4(1)	4(1)	0.2	13%
Q9NP64	ZCCHC17	27894	51	2(1)	2(1)	0.12	11%
Q96EY5	MVB12A	29107	49	2(2)	2(2)	0.24	10%
Q9BZX2	UCK2	29452	47	3(1)	3(1)	0.11	15%

Accession	Description	Mass	Score	Matches	Sequences	emPAI	Coverage
Q07955	SRSF1	27842	46	3(1)	2(1)	0.12	8%
P07339	CTSD	45037	46	6(1)	5(1)	0.24	14%
P09497	CLTB	25289	44	1(1)	1(1)	0.13	3%
Q08378	GOLGA3	167765	43	4(1)	2(1)	0.02	1%
Q7L576	CYFIP1	146742	42	1(1)	1(1)	0.02	1%
P02649	APOE	36246	42	4(1)	2(1)	0.09	7%
P02647	APOA1	30759	41	4(1)	2(1)	0.11	10%
Q6IAN0	DHRS7B	35382	41	1(1)	1(1)	0.09	4%
Q96CT7	CCDC124	25820	41	3(1)	3(1)	0.13	19%
Q9P0L0	VAPA	28103	41	1(1)	1(1)	0.12	5%
P04004	VTN	55069	38	1(1)	1(1)	0.06	3%
Q9UIV1	CNOT7	33066	38	3(1)	2(1)	0.33	10%
Q14966	ZNF638	221914	38	7(1)	6(1)	0.01	2%
P15924	DSP	334021	37	24(1)	11(1)	0.01	4%
Q13151	HNRNPA0	30993	35	2(1)	2(1)	0.23	8%
P50897	PPT1	34627	35	1(1)	1(1)	0.1	4%
Q9HBM6	TAF9B	27718	35	1(1)	1(1)	0.12	3%
Q6UXN9	WDR82	35456	34	2(1)	2(1)	0.09	8%
Q15365	PCBP1	37987	34	1(1)	1(1)	0.09	3%
P78417	GSTO1	27833	33	2(1)	2(1)	0.12	7%
Q9Y5Y2	NUBP2	29206	31	2(0)	2(0)	0.11	6%
P08574	CYC1	35741	31	1(0)	1(0)	0.09	3%
P08670	VIM	53676	30	3(0)	3(0)	0.06	6%
O60762	DPM1	29673	30	1(0)	1(0)	0.11	3%
Q15029	EFTUD2	110336	28	4(0)	4(0)	0.03	5%
Q9H324	ADAMTS10	124559	26	2(0)	1(0)	0.03	0%
Q7L523	RRAGA	36942	26	3(0)	3(0)	0.09	13%
P48730	CSNK1D	47585	26	3(0)	2(0)	0.07	3%
Q9Y315	DERA	35494	23	1(0)	1(0)	0.09	3%
P78363	ABCA4	258232	23	11(0)	3(0)	0.01	1%
Q16831	UPP1	34653	22	3(0)	2(0)	0.1	4%
Q674X7	KAZN	86754	21	5(0)	2(0)	0.04	2%
Q06323	PSME1	28876	19	2(0)	2(0)	0.12	8%
P49411	TUFM	49852	19	1(0)	1(0)	0.07	4%
P0C0L4	C4A	194261	19	1(0)	1(0)	0.02	0%
Q8NB66	UNC13C	252693	18	2(0)	1(0)	0.01	0%
Q8NA03	FSIP1	66593	18	2(0)	2(0)	0.05	4%
P06733	ENO1	47481	17	3(0)	3(0)	0.07	10%
O75095	MEGF6	174757	17	5(0)	2(0)	0.02	1%
P02652	APOA2	11282	16	1(0)	1(0)	0.3	22%
P05156	CFI	68102	16	1(0)	1(0)	0.05	1%
P27816	MAP4	121443	16	4(0)	2(0)	0.03	2%

Accession	Description	Mass	Score	Matches	Sequences	emPAI	Coverage
Q2M243	CCDC27	75821	14	4(0)	2(0)	0.04	4%

Table S2

**Primers for dual-luciferase reporter assay.**	
**Primer**	**Sequences (5'-3')**
Promoter of SerRS (Forward)	TCAGGTACCAGTAGAGACGGGATTTTGTCATG
Promoter of SerRS (Reverse)	TCACTCGAGCAAATCCAGATCCAGCACCATCTTC

**Primers for shRNAs for gene knockdown.**	
**Primer**	**Sequences (5'-3')**
SerRS-sh1	AAAAGGCATAGGGACCCATCATTGATTG<br>GATCCAA TCAATGATGGGTCCCTATGCC
NCOR2-sh1	AAAAGCAGCCTGGTGCAGATCATCTTTG<br>GATCCAAAGATGATCTGCACCAGGCTGC
NCOR2-sh2	AAAAGCTGAGTGCGTCCTCTATTACTTG<br>GATCCAAGTAATAGAGGACGCACTCAGC
NCOR2-sh3	AAAAGGTCAAGTCCAAGAAGCAAGATTGG<br>ATCCAATCTTGCTTCTTGGACTTGACC

**Primers for quantitative RT–PCR.**	
**Primer**	**Sequences (5'-3')**
Human SerRS (Forward)	AAGAAAGCAGCAGCAAGAGACG
Human SerRS (Reverse)	CATGCGAGGAGACAGGAACATC
Human VEGFA (Forward)	GAGGGCAGAATCATCACGAAG
Human VEGFA (Reverse)	TGTGCTGTAGGAAGCTCATCTCTC
Human β-actin (Forward)	CGTCACCAACTGGGACGA
Human β-actin (Reverse)	ATGGGGGAGGGCATACC
Human NCOR2 (Forward)	AAGCCTTACATGCCTCTGGGA
Human NCOR2 (Reverse)	TCATGGACTTTGGTGACCTGG
Human HOXB1 (Forward)	CCAGCTACGGGCCTTCTCAG
Human HOXB1 (Reverse)	CAGCATAGGCCGGTGCAAAG
Human PCK1 (Forward)	CCAAGAAGGTGGGGACGTTCA
Human PCK1 (Reverse)	TCCTGCCACATAAGCCTCACC
Human UCP1 (Forward)	GAGGGTGGGTAGGAGGGGAC
Human UCP1 (Reverse)	CAAGCACGCCGCTATTCCAG
Mouse SerRS (Forward)	AAGGGGGTGTAGATTGGAGTGT
Mouse SerRS (Reverse)	ACCTGGTGGTGATGGTAGATGG
Mouse VEGFA (Forward)	GTCCGATTGAGACCCTGGTG
Mouse VEGFA (Reverse)	TTGACCCTTTCCCTTTCCTCG
Mouse β-actin (Forward)	GGCTGTATTCCCCTCCATCG
Mouse β-actin (Reverse)	GCACAGGGTGCTCCTCAG

Zebrafish SerRS (Forward)	TGCGACCACTCGTGTCATCT
Zebrafish SerRS (Reverse)	GAGACTGTGGAGGGCGTGTC
Zebrafish VEGFA (Forward)	GGCTCTCCTCCATCTGTCTGC
Zebrafish VEGFA (Reverse)	CAGTGGTTTTCTTTCTTTGCTTTG
Zebrafish β-actin (Forward)	TCACCACCACAGCCGAAAGAG
Zebrafish β-actin (Reverse)	GTCAGCAATGCCAGGGTACAT

Primers for ChIP assay.

Primer	Sequences (5'-3')
RARE1 (Forward)	AGCTATGTCACACCCTCATCTCAG
RARE1 (Reverse)	AAAGCCTCACTTCCAATGCCTTC
RARE2 (Forward)	AGAGACGGGATTTTGTCATGTTGG
RARE2 (Reverse)	TGGCTAATGCCTGTAATCCTAACACT

Primers for NCOR2 SANT2 expression construct.

Primer	Sequences (5'-3')
NCOR2 SANT2 (Forward)	TCAGGATCCCAGCAGCAGCAGCAGCCCATG
NCOR2 SANT2 (Reverse)	TCACTCGAGCTAGTGCTGCTGCAAGATCTCATCGA
